# *De novo* cysteine biosynthesis in *Pseudomonas aeruginosa*: Characterization of the two main cysteine synthase isoforms

**DOI:** 10.1016/j.isci.2025.114304

**Published:** 2025-12-02

**Authors:** Rebecca Martedì, Jole Maria Lucia D’Angelo, Giulia Sassi, Marialaura Marchetti, Sarah Hijazi, Riccardo Percudani, Stefano Bettati, Barbara Campanini, Emanuela Frangipani

**Affiliations:** 1 Department of Biomolecular Sciences, University of Urbino Carlo Bo, Urbino, PU, Italy; 2 Department of Food and Drug, University of Parma, Parma, PR, Italy; 3 Department of Chemistry, Life Sciences and Environmental Sustainability, University of Parma, Parma, PR, Italy; 4 Department of Medicine and Surgery, University of Parma, Parma, PR, Italy; 5 Interdepartmental Center Biopharmanet-TEC, University of Parma, Parma, PR, Italy

**Keywords:** Biosynthesis, Microbial genetics, Molecular microbiology

## Abstract

Most bacteria synthesize L-cysteine *via* the reductive sulfate assimilation pathway, which is absent in humans and thus a promising source of antibiotic targets. Despite its relevance, this pathway remains poorly studied in *Pseudomonas aeruginosa*, a major antimicrobial resistance (AMR)-associated pathogen.

We have identified the two main isoforms of cysteine synthase in *P. aeruginosa* (PA2709 and PA0932), which are pyridoxal 5′-phosphate-dependent enzymes that enable bacterial growth in minimal medium supplemented with either sulfate or thiosulfate. PA2709 is a classical *O*-acetylserine (OAS) sulfhydrylase, using bisulfide as a sulfur source. PA0932 also shows an OAS-dependent *S*-sulfocysteine synthase activity. Deletion of either one of the two genes does not lead to cysteine auxotrophy, which is reached only with the double deletion mutant. Interestingly, in the presence of thiosulfate as the only sulfur source, PA0932 displays a cysteine bradytrophic phenotype, suggesting the activation of an alternative sulfur assimilation pathway under these conditions.

## Highlights

• Sulfate assimilation pathway, absent in humans, provides new antibiotic targets
• Two such targets are the cysteine synthases PaCysK and PaCysM of *P. aeruginosa*
• The physiological role and substrate specificity of PaCysK and PaCysM were elucidated
• Some functions of PaCysK and PaCysM may not be associated with cysteine production

## Graphical abstract

**Figure:**
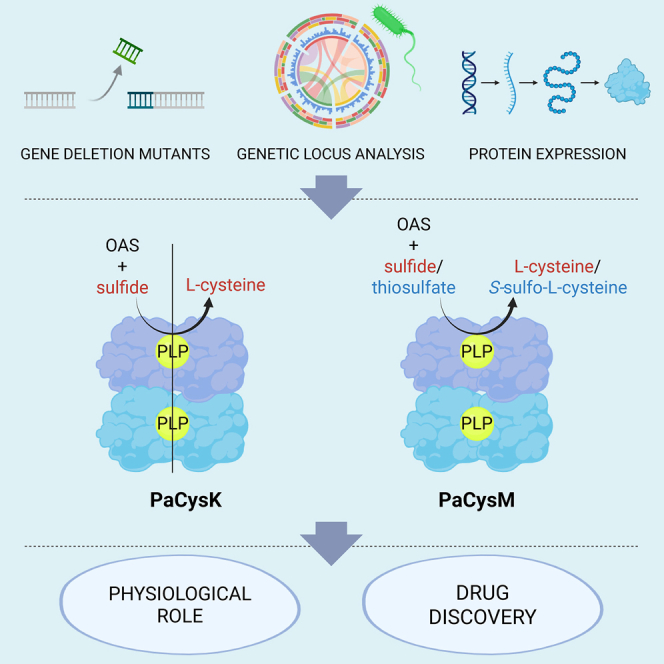

## Introduction

**Scheme 1:**
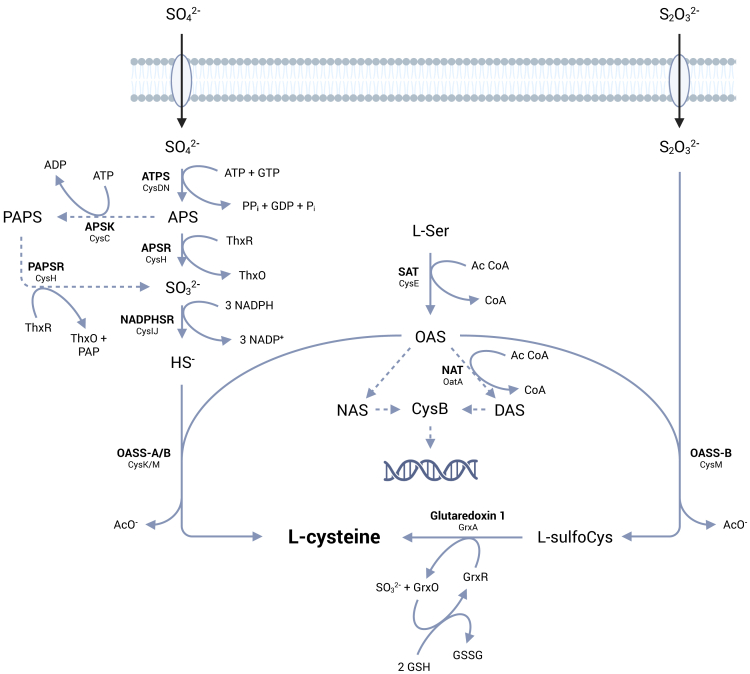
General scheme for L-cysteine biosynthesis in model organisms *E. coli* and *S.* Typhimurium Below the enzymatic activities, shown in bold, the corresponding protein names as annotated in the *E. coli* genome are reported (ATPS, ATP sulfurylase; APSK, APS kinase; PAPSR, 3′-phosphoadenosine-5′-phosphate reductase; APSR, APS reductase; NADPHSR, sulfite reductase [NADPH-dependent]; NAT, *N*-acetyltransferase; SAT, serine acetyltransferase; OASS-A, *O*-acetylserine sulfhydrylase A; OASS-B, *O*-acetylserine sulfhydrylase B). Modified from Kredich, 1992.^1^

The need of bacteria to adapt to different niches has pushed the evolution of *de novo* biosynthetic pathways, some of which are completely absent in mammals. One example of such metabolism is the assimilation of inorganic sulfur into cysteine, a key intermediate that enables the incorporation of sulfur in various metabolites that harness its unique chemical properties. *De novo* cysteine biosynthesis is performed through the reductive sulfate assimilation pathway (RSAP) that activates sulfate (SO_4_^2−^) into bisulfide (HS^−^), which is ultimately incorporated into *O*-acetylserine (OAS), by the enzyme *O*-acetylserine sulfhydrylase (OASS, Scheme 1).

**Scheme 2:**
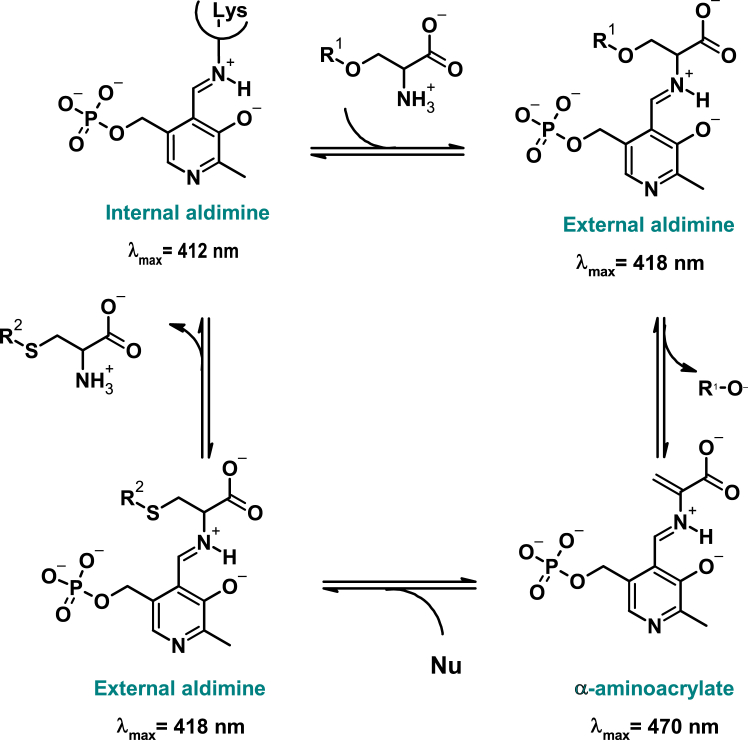
General reaction mechanism of cysteine synthases The scheme shows the reaction using either OAS (R^1^ = CH_3_CO) or OPS (R^1^ = PO_3_^2−^) as first substrate and either bisulfide or thiosulfate (R^2^ = H/SO_3_^2−^) as nucleophile (Nu) in the second half-reaction. The names of the intermediates formed during the catalytic cycle are indicated in green, together with the wavelengths of their maximal absorption. Only the ketoenamine tautomer of the intermediates is shown, the enolimine tautomer (Figure S1) absorbs at around 330 nm for all intermediates.

Sulfate is the most abundant form of inorganic sulfur in the environment, while thiosulfate (S_2_O_3_^2−^) is more prominent under less oxidizing conditions.^2^ In bacteria, thiosulfate enters cysteine biosynthesis bypassing the reductive assimilation, being directly incorporated into *S*-sulfocysteine by specialized OASSs.^3^^,^^4^ *S*-sulfocysteine can subsequently be converted to cysteine by the action of glutaredoxins.^5^ This very general framework for bacterial cysteine biosynthesis has been the subject of interesting evolutionary divergence. Certain microorganisms, such as *Mycobacterium tuberculosis*, can utilize the serine biosynthesis intermediate *O*-phosphoserine (OPS) in place of OAS^6^^,^^7^ or a protein-bound thiocarboxylate (CysO-SH) as the sulfide donor.^8^ In some cases, bacteria also possess an active reverse transsulfuration pathway, in which cysteine is synthesized from methionine.^9^^,^^10^^,^^11^^,^^12^^,^^13^^,^^14^^,^^15^ In the most conserved architecture of the RSAP, the final step is catalyzed by either CysK or CysM. These pyridoxal 5′-phosphate (PLP)-dependent enzymes catalyze a ping-pong reaction where OAS undergoes a β-elimination with release of acetate and accumulation of the α-aminoacrylate intermediate, which is then attacked by either bisulfide or thiosulfate (Scheme 2) to give the final product. The orthologs from *Escherichia coli* and *Salmonella* Typhimurium are very well characterized both structurally and enzymatically.^16^^,^^17^^,^^18^^,^^19^^,^^20^^,^^21^^,^^22^^,^^23^^,^^24^^,^^25^ Both isoforms have a homodimeric structure, with the active sites located distally from the dimer interface and within a crevice formed by the N-terminal and C-terminal domains. CysK, but not CysM, can form with the preceding enzyme in the pathway, serine acetyltransferase (*i.e.,* SAT/CysE), a bienzymatic complex whose functional and regulatory roles are still poorly understood.^26^^,^^27^^,^^28^

Cysteine biosynthesis is subject to tight regulatory control to maintain intracellular sulfur homeostasis and hamper its accumulation that may lead to increased sensitivity to H_2_O_2_ by fueling the Fenton reaction.^29^ In *E. coli*, accumulation of cysteine negatively regulates the biosynthetic pathway through feedback inhibition of SAT, with an IC_50_ of 0.18 μM.^28^ On the other hand, sulfur limitation leads to the accumulation of OAS that is converted to either *N*-acetylserine, by a spontaneous reaction, or to *N,O*-diacetylserine by a reaction catalyzed by OatA *N*-acetyltransferase. Both molecules have been reported to be inducers of the cysteine biosynthetic operon, acting through the CysB transcriptional regulator.^1^^,^^30^

Cysteine biosynthesis plays a central role in bacterial physiology, extending beyond sulfur assimilation to impact many cellular functions, some of which are linked to redox homeostasis, virulence, and antibiotic resistance (recently reviewed in Tikhomirova, 2024^31^). These aspects have been extensively studied in *E. coli*, *S.* Typhimurium, and *M. tuberculosis*,^32^^,^^33^^,^^34^^,^^35^^,^^36^^,^^37^ and some recent works by our and other groups underlie the exploitability of this pathway as a source of targets for the development of antibiotic enhancers.^38^^,^^39^^,^^40^^,^^41^^,^^42^^,^^43^ The ability of *P. aeruginosa* to grow on inorganic sulfur sources (*e.g.*, sulfate and thiosulfate) was documented nearly five decades ago.^4^^,^^44^ However, sulfur assimilation pathways in this microorganism have received limited attention, despite evidence indicating a requirement for cysteine biosynthesis during chronic lung infections in cystic fibrosis patients,^45^ where *P. aeruginosa* is the predominant bacterial pathogen and leading cause of mortality.^46^^,^^47^^,^^48^ These metabolic adaptations highlight the importance of RSAP for sustaining a successful chronic pulmonary infection and warrant further investigations to elucidate the role of the enzymatic components involved in the pathway.

In this study, we provide a comprehensive characterization of the two primary cysteine synthases in *P. aeruginosa* (CysK and CysM), through an integrated approach involving recombinant protein expression/purification, detailed biochemical analyses, and the construction of *P. aeruginosa* deletion mutants lacking the *cysK* and *cysM* genes.

## Results

### Analysis of the genetic loci and of coding sequences

A preliminary interrogation of the *P. aeruginosa* genome database (PAdb, https://www.pseudomonas.com/) allowed the identification of genes encoding proteins potentially involved in the RSAP. However, most entries were only annotated based on sequence homology to proteins of known function but lacked functional characterization. CysK and CysM amino acid sequences from *E. coli* (Uniprot Entries P0ABK5, P16703) have been used to retrieve putative orthologs in *P. aeruginosa* using the UniProt BLASTp tool. Four putative OASSs enzymes have been identified (Table 1); two of them (PA2709 and PA0932), encoded by genes annotated as *cysK* and *cysM*, respectively, showing the highest similarity to *E. coli* orthologs (≃ 70% AA identity), were chosen for subsequent experiments.

**Table 1:** Summary of the sequences identified as coding for cysteine synthase in *P. aeruginosa*

Enzyme	UniProt accession number	PAdb ID	Best match in *E. coli* K12^a^	Percent identity
Cysteine synthase A	Q9I0D3	PA2709	CysK	70.8
Cysteine synthase B	Q9I526	PA0932	CysM	70.0
Probable cysteine synthase	Q9I211	PA2104	CysK	34.3
Conserved hypothetical protein	Q9I4R3	PA1061	CysM	28.5

a The best hits in *E. coli* K12 were identified using the UniProt BLASTp tool with *P. aeruginosa* proteins as queries.

**Figure 1:**
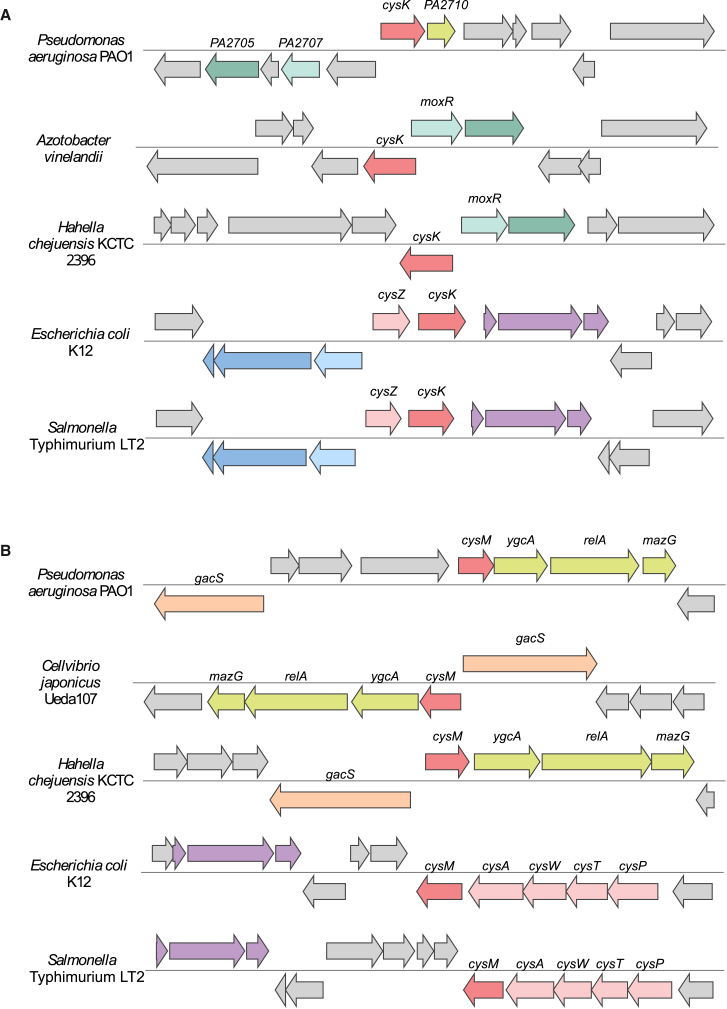
c*ysK* and *cysM* genetic loci Contigs of five representative microbial genomes are aligned around orthologs of PA2709 (putative *cysK*, A, in red) and PA0932 genes (putative *cysM*, B, in red). Genes are represented by arrows indicating the direction of transcription, and orthologs are depicted in the same color. Other genes are shown in light gray. (A) In *P. aeruginosa*, *A. vinelandii*, and *H. chejuensis*, *cysK* is located near PA2707 (light green) and PA2710 (dark green). In *P. aeruginosa* alone, a non-conserved gene from the lysine transporter family (PA2710, in yellow) is found adjacent to *cysK.* In *E. coli* and *S.* Typhimurium, *cysK* is close to the sulfate transporter (*cysZ* in pink) and flanked by phosphoenolpyruvate-dependent sugar phosphotransferase system operon (in violet), the *ligA-ypeB* cluster (in blue), and the zip gene (in light blue). (B) In *P. aeruginosa*, *C. japonicus*, and *H. chejuensis*, *cysM* is adjacent to genes involved in (p)ppGpp metabolism (*relA, mazG*) and 23S rRNA uridine methylation (*ygcA*, in yellow) as well as the adaptive response histidine kinase gene (*gacS*, in light orange). In *E. coli* and *S.* Typhimurium, *cysM* is part of the *cysPTWAM* operon for sulfate transport (in pink). Also conserved is the phosphoenolpyruvate-dependent sugar phosphotransferase system (in violet).

An analysis carried out using the RAST server was performed to investigate the genomic context of PA2709 and PA0932. The genetic locus of PA2709 (putative *cysK*, Figure 1A) is not well conserved among bacteria, and most of the surrounding genes code for hypothetical proteins. However, some genes within the region, such as PA2707 and PA2705, are also conserved among different species of Gammaproteobacteria. PA2707 is predicted to be a MoxR-like AAA+ ATPase, while PA2705 and its orthologs are unannotated. The unconserved PA2710 gene, located near *cysK*, is predicted to code for a LysE family translocator. Notably, the *eamB* gene in *E. coli* also belongs to the LysE family and functions as an exporter of OAS and cysteine.^49^ In contrast, in other species such as *E. coli* and *S.* Typhimurium, the *cysK* locus is well conserved and is located adjacent to the *cysZ* transporter gene, which mediates sulfate uptake. A search in STRING confirms the overall lack of conservation of the genetic locus, as no high-confidence neighboring genes are observed.

The genetic locus of PA0932 (putative *cysM*, Figure 1B) is overall better conserved than PA2709, particularly with respect to those of *Oceanospirillales* and *Cellvibrionales* (Gammaproteobacteria). *cysM* is located upstream to the *ygcA* gene, predicted to code for the methyltransferase RlmD that catalyzes the formation of 5-methyl-uridine at position 1939 (U1939) in 23S rRNA. Interestingly, among the annotated genes, also *relA* and *mazG* are consistently present in different species, on the same DNA strand. RelA is a GTP diphosphokinase that synthesizes alarmone in response to harsh environmental factors, and MazG is a nucleoside triphosphate pyrophosphohydrolase that may degrade the alarmone and contribute to tuning the stringent response. A possible association was proposed between *ygcA* and RelA-dependent stringent response during amino acid starvation,^50^ considering that RelA becomes activated upon the interaction with 23S rRNA in the A site of the ribosome and that the loop containing U1939 tucks in the major groove at the end of the acceptor stem of the A-site tRNA. A search in STRING also supports *ygcA* and *relA* as conserved genes in the *cysM* genetic locus, with high and medium confidence scores, respectively.

**Figure 2:**
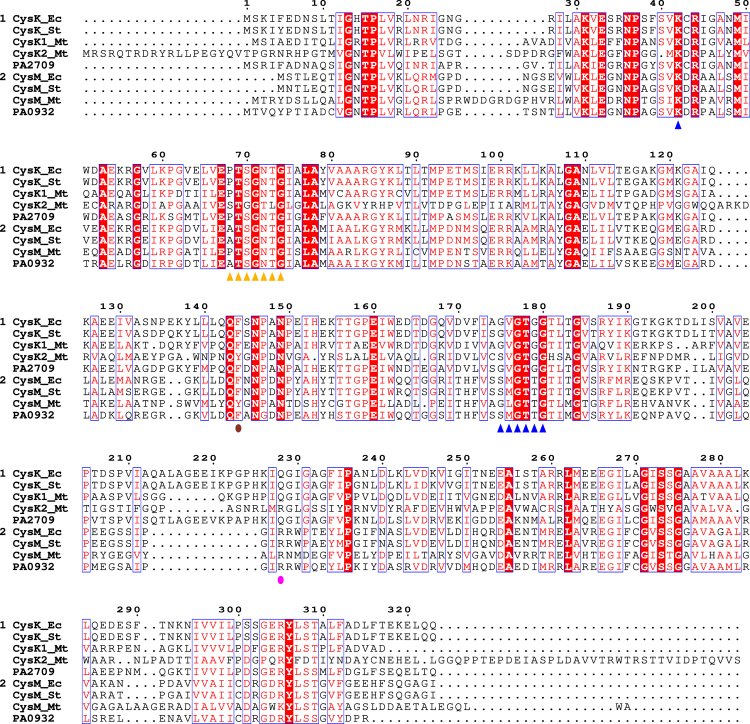
Sequence conservation of CysK and CysM proteins Multiple alignment of CysK sequences from *E. coli* (Ec), *S.* Typhimurium (St), *M. tuberculosis* (Mt), and *P. aeruginosa* (group 1), and CysM sequences from the same species (group 2). Identical residues have a red background, and residues with similar physicochemical properties are shown in red. Active site residues involved in the binding of PLP (purple triangles) and amino acidic substrates (orange triangles) are according to.^18^^,^^19^^,^^20^^,^^21^^,^^25^^,^^51^ Discriminant residues for OPS binding and thiosulfate binding^52^^,^^53^ are depicted by brown and pink circles, respectively.

The translated sequences of PA2709 and PA0932 were aligned with the CysK and CysM sequences from *E. coli*, *S.* Typhimurium, and *M. tuberculosis* (Figure 2). PA2709 showed the greatest percent identity (Figure S2) with CysK proteins from *E. coli* (71.4%), *S.* Typhimurium (71.1%), and *M. tuberculosis* (56.0%). PA0932 has the highest percent identity with CysM proteins from *E. coli* and *S.* Typhimurium (68.8%). However, it is more similar to PA2709 (43.4%) and CysK proteins from *E. coli* and *S.* Typhimurium than to the CysM protein from *M. tuberculosis*.

Interestingly, most functionally important residues^18^^,^^19^^,^^20^^,^^25^^,^^51^^,^^52^^,^^53^^,^^54^ are conserved, indicating that both proteins likely bind the PLP cofactor and also bind amino acidic substrates. Indeed, the putative catalytic Lys (blue triangle in Figure 2), Lys44 in PA2709 and Lys45 in PA0932, is conserved and should bind PLP to form the internal aldimine. Residues 177–182 in PA2709 and 176–181 (blue triangles in Figure 2) could form the so-called phosphate binding cup, *i.e.,* a conserved structure in PLP-dependent enzymes that anchors PLP to the active site by specific interactions with the phosphate group. Residues 70–76 in PA2709 and 71–77 in PA0932 (orange triangles) are located in a conserved stretch of residues that, in *S.* Typhimurium, form the substrate binding loop, i.e., residues that once engaged in specific interactions with the substrate carboxylic moiety, trigger a conformational change in the active site leading to its closure to protect reactive catalytic intermediates.^25^ One final interesting feature emerges from the conservation pattern at positions 145/146 (brown circle) and 229/214 (purple circle): both proteins have a Phe at position 145/146 indicating a preference for OAS over OPS (orthologs that use OPS usually have a Tyr at this position^53^); PA2709 has a Gln at position 229 while PA0932 has an Arg at position 214, an indication that the latter might be able to use thiosulfate in addition to bisulfide.^52^ PA2709 also has a 13 residues insertion (Lys222-Phe235), which is absent in PA0932, that, in *E. coli, S.* Typhimurium, and *Arabidopsis thaliana,* forms the β8A–β9A loop, a conserved region of the CysK sequence situated near its active site. This loop contains three residues (K222, H226, and K227) that are essential for CysE binding, as their mutation disrupts the CysK-CysE complex.^26^^,^^55^^,^^56^ The sequence analysis allows to predict that the two proteins are likely PLP-dependent enzymes with functional properties close to those of CysK and CysM.

### Recombinant protein expression and biophysical characterization

**Figure 3:**
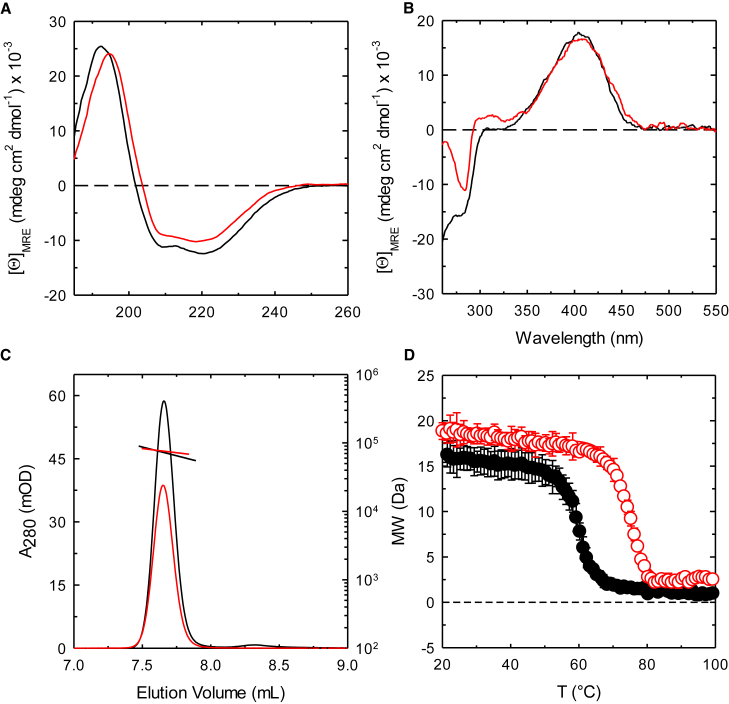
Biophysical characterization of PA2706 (black symbols) and PA0932 (red symbols) (A) Far-UV circular dichroism spectra were collected in a 20 mM potassium phosphate buffer, pH 7.0, at 20 °C on solutions containing 4 μM protein (monomer concentration). (B) Near-UV circular dichroism spectra were collected in a 20 mM potassium phosphate buffer, pH 7.0, at 20 °C on solutions containing 60 μM protein (monomer concentration). (C) Oligomeric state determination. Size-exclusion chromatography (SEC) chromatograms obtained from an Agilent AdvanceBio 300 Å column coupled to UV-vis and dual-angle light scattering detectors. The proteins were analyzed at a concentration of 1 mg/mL. Data shown are a representative run of two independent experiments. (D) Thermal denaturation collected at 195 nm on solutions containing 4 μM protein in 20 mM potassium phosphate buffer, pH 7.0. Lines through data points are the fitting to Equation 1 with T_m_ = 60.0 ± 0.1 °C for PA2706 and T_m_ = 74.6 ± 0.2 °C for PA0932. Each value is the average of two independent experiments ± standard deviation.

PA2709 and PA0932 have similar, but not superimposable, circular dichroism spectra in the far-UV region (Figure 3A). The deconvolution with DichroWeb^57^ gave the secondary structure content shown in Table S1, which is in agreement with data obtained from three-dimensional structures of CysK and CysM orthologs from *S.* Typhimurium (1OAS and 2JC3^19^^,^^20^). The near-UV spectra are almost superimposable and suggest the binding of the PLP cofactor at the active site of both proteins (Figure 3B). Indeed, PLP is not dichroic when free in solution and the measurable signal around 400 nm indicates that the cofactor is bound within an asymmetric site. Size exclusion chromatography indicates that both enzymes are dimeric in solution with an estimated mass of 71 kDa for PA2709 (calculated mass for a dimer: 69 kDa) and of 75 kDa for PA0932 (calculated mass for a dimer: 65 kDa) (Figure 3C). The thermal stability was assessed on the temperature ramps collected using the circular dichroism signal at 195 nm. Interestingly, PA0932 shows a T_m_ of about 74 °C, which is 15 °C higher than the T_m_ measured for PA2709 (Figure 3D).

**Figure 4:**
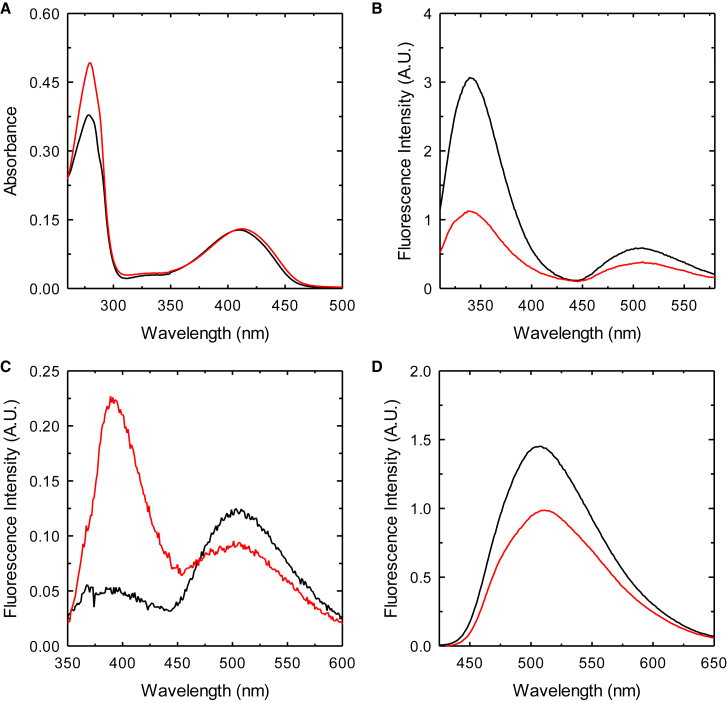
Spectroscopic characterization of PA2709 (black lines) and PA0932 (red lines) (A) Absorption spectra collected on a 20 μM enzyme solution in buffer H (100 mM HEPES, pH 7). (B) Fluorescence emission spectra for excitation at 298 nm, slits = 5 nm. (C) Fluorescence emission spectra for excitation at 330 nm, slits = 5 nm. (D) Fluorescence emission spectra for excitation at 412 nm, slits = 5 nm. Fluorescence spectra were collected on a 3 μM protein solution in buffer H.

The UV-vis absorption spectra of PA2709 and PA0932 both show two peaks, one centered at 280 nm due to the absorption of aromatic residues (PA0932 has 9 tyrosine residues compared with 5 of PA2709) the other centered at 411 and 413 nm for PA2709 and PA0932, respectively (Figure 4A).

The band in the visible region is due to the absorption of the PLP cofactor bound as an internal aldimine to the catalytic lysine (Scheme 1) that, based on sequence alignments in Figure 2, should be Lys44 for PA2709 and Lys45 for PA0932. The internal aldimine of PLP can exist in two tautomeric forms, the enolimine, which absorbs at about 330 nm, and the ketoenamine, which absorbs at about 412 nm (Figure S1). The last tautomer, usually favored by polar active site environments, is the prevailing one in the case of both enzymes. The fluorescence emission of the protein upon the selective excitation at 298 nm of the Trp residues (Trp53/164 for PA2709 and Trp163/216 for PA0932) leads to a structured emission with a major peak centered at 340 nm, due to the direct emission of Trps, and a minor peak at about 505 nm due to the energy transfer between the Trp(s) and the PLP cofactor (Figure 4B). Direct excitation of the enolimine tautomer of the cofactor at 330 nm leads to an emission spectrum with two peaks: one centered at 390 nm due to the direct emission of the enolimine tautomer, the other one centered at 505 nm, which is due to the emission of the ketoenamine tautomer that forms upon excited state proton transfer.^58^ The relative intensity of the two peaks is inverted for PA0932 and PA2709, with the emission at 390 nm being higher for PA0932 and the one at 505 higher for PA2709 (Figure 4C). The emission peak upon direct excitation of the ketoenamine tautomer at 412 nm is slightly redshifted for PA0932 (511 *vs*. 507 nm), signaling a slightly more polar environment of the cofactor (Figure 4D).

### *In vitro* enzymatic activity of PA2709 and PA0932

**Figure 5:**
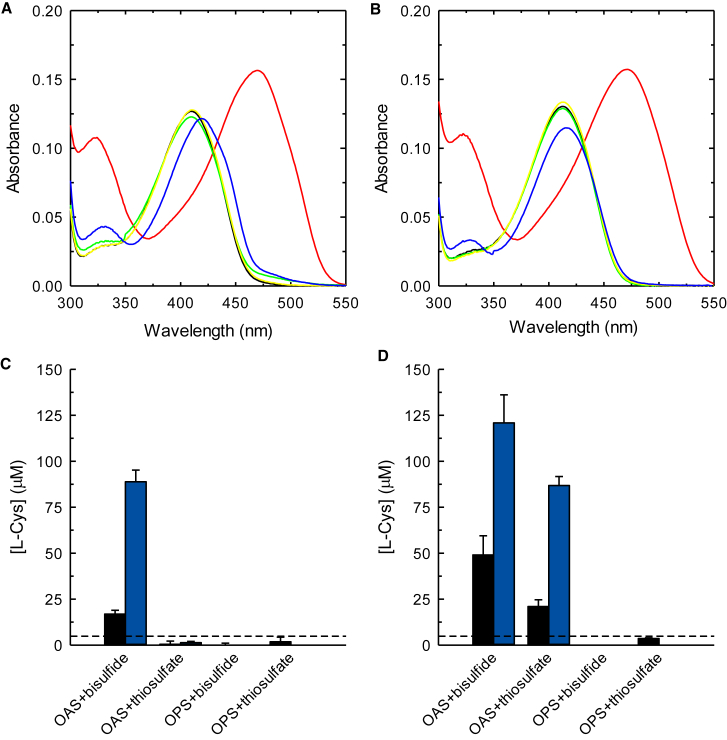
Reactivity of PA2709 and PA0932 with amino acids and sulfur donors (A and B) Absorption spectra of either 20 μM PA2709 (A) or 20 μM PA0932 (B) in the absence of added amino acids (black lines) and in the presence of 10 mM OAS (red lines), 10 mM OPS (green lines), 10 mM L-Ser (yellow lines), or 10 mM L-Cys (blue lines) in buffer H. (C and D) Production of L-Cys by either PA2709 (C) or PA0932 (D) in the presence of different substrate combinations at 2 min (black bars) and 10 min (blue bars) after reaction triggering. Each bar is the average of two independent experiments ± standard deviation. The concentrations used are as follows: 3.1 nM enzyme, 10 mM OAS, 10 mM OPS, 0.1 mM HS^−^, 0.2 mM thiosulfate in buffer H. The product of thiosulfate and OAS is *S*-sulfocysteine that, under the acidic conditions of the ninhydrin assay employed,^62^ is promptly hydrolyzed to L-Cys. The dashed line represents the detection limit of the assay.

The reactivity of PA2709 and PA0932 with potential substrates has been investigated first by absorption spectroscopy, exploiting the signal of the cofactor that reports on the various intermediates of the catalytic cycle (Scheme 2; Figures 5A and 5B). Addition of 10 mM OAS leads to the disappearance of the absorption band of the internal aldimine and to the appearance of two bands, centered at 323 nm and 470 nm, attributed to the enolimine and ketoenamine tautomers of the *α*-aminoacrylate. Differences in the fluorescence emission spectra upon excitation at 298 nm (Figure S3) of the two enzymes upon reaction with OAS signal differences in the active site microenvironment of this intermediate. Addition of either OPS or L-Ser does not lead to spectroscopic changes, indicating that these two amino acids are not substrates for the first half-reaction of the enzyme. The lack of reactivity is confirmed by the fluorescence emission spectra upon excitation at 298 nm (Figure S3). Conversely, addition of 10 mM L-Cys leads to a redshift of the band at 412 nm and to an increase of the band at 330 nm, indicative of the formation of an adduct with the cofactor, either a lanthionine, as observed for other fold-type II PLP-dependent enzymes,^59^^,^^60^ or, most likely, the external aldimine with cysteine. The fluorescence emission spectra upon excitation at 298 nm confirm the formation of an external aldimine that is typically associated with a large increase in the fluorescence emission band at 505 nm, as already observed for other OASS orthologs.^20^ The stability of the α-aminoacrylate was assessed monitoring for up to 60 min the absorption spectra after addition of either 100 μM or 10 mM OAS (Figure S4). In the presence of 10 mM OAS the intermediate is stable, and no differences are observed between the two enzymes (Figure S4C, dotted line). Conversely, when 100 μM OAS is used, the stability of α-aminoacrylate formed by PA2709 is not affected, while this intermediate decays after 10 min in the case of PA0932. This different behavior has been previously reported for the CysK and CysM isozymes of *S.* Typhimurium^20^^,^^22^^,^^61^ and has been attributed to the different relative efficiency of the hydrolytic side reaction that converts the α-aminoacrylate intermediate to pyruvate and ammonia. This result is therefore suggestive of defined catalytic properties that would confirm the attribution of PA2709 to a CysK isoform and PA0932 to a CysM isoform. Keeping the concentration of OAS at 100 μM the two main sulfur donors reported in the literature for cysteine synthases were tested, namely, bisulfide and thiosulfate (Figure S5). In the case of PA2709, the addition of bisulfide leads to the instantaneous disappearance of the α-aminoacrylate and the formation of the internal aldimine, indicative of an efficient second half-reaction. No reaction was measured with thiosulfate on PA2709 (data not shown). On the other hand, PA0932 can react with both bisulfide, although at a slower rate compared to PA2709, and thiosulfate. These data further suggest a cysteine synthase activity for both isoforms and an additional *S*-sulfocysteine synthase activity for PA0932 only. This activity was indeed confirmed by measuring the amount of L-Cys formed in the presence of all the possible substrate couples: OAS+bisulfide, OAS+thiosulfate, OPS+bisulfide, OPS+thiosulfate (Figures 5C and 5D). PA2709 is reactive only with the OAS+bisulfide substrate couple, while PA0932 is reactive with both OAS+bisulfide and OAS+thiosulfate couples.

**Figure 6:**
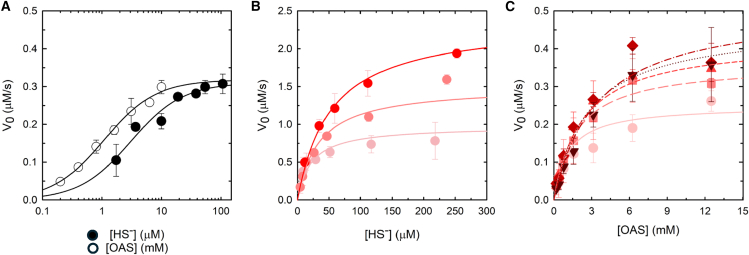
Determination of the catalytic parameters for PA2709 and PA0932 (A) Initial velocity as a function of bisulfide concentration (closed dots) in the presence of 10 mM OAS and as a function of OAS concentration (open dots) in the presence of 60 μM bisulfide for PA2709 in buffer H at 25°C. Each value is the average of two independent experiments ± standard deviation. Lines through data points represent the fitting to Equation 3 with the parameters reported in Table 2 (PA2709). (B) Initial velocity as a function of bisulfide concentration for PA0932 in buffer H at 25 °C in the presence of 5 mM (pink dots), 10 mM (light red dots), and 30 mM (red dots) OAS. Each value is the average of two independent experiments ± standard deviation. Lines through data points represent the fitting to Equation 4 with the parameters reported in Table 2. (C) Initial velocity as a function of OAS concentration for PA0932 in the presence of 0.1 mM (light pink dots), 0.2 mM (pink squares), 0.3 mM (light red triangles), 0.4 mM (red diamonds), and 0.6 mM (dark red triangles) thiosulfate in buffer H at 25°C. Each value is the average of two independent experiments ±standard deviation. Lines through data points represent the global fitting to Equation 5 with the parameters reported in Table 2.

These preliminary experiments prompted the determination of catalytic parameters for the two enzymes. From literature data on paralogs, we assumed that the Michaelis constants for both bisulfide and thiosulfate are in the low micromolar range, with bisulfide reported to have K_m_ values as low as 3 μM.^22^^,^^63^^,^^64^ This property makes the use of the ninhydrin assay^65^ inappropriate to measure catalytic parameters (detection limit 4.9 μM, see STAR Methods) and we thus set up a method, based on seminal works by Cook and collaborators,^66^^,^^67^ that exploits the continuous monitoring in the reaction mixture of bisulfide disappearance by an ion-selective electrode. The kinetics showed a linear phase at 50 μM bisulfide and 10 mM OAS that could be fitted to calculate the initial rate (Figure S6). No burst or lag phases were apparent. The reaction rate was linear with respect to enzyme concentration within 1.5 and 6 nM enzyme concentration for both PA2709 and PA0932 (Figure S6). The assay was therefore used to measure the catalytic parameters for one substrate keeping the other constant for both enzymes, using bisulfide as nucleophile (Figure 6). The dependence of the initial velocity on bisulfide concentration was measured at 10 mM OAS for PA2709 and PA0932, considering that the paralogs from *S.* Typhimurium have a comparable K_m_ of about 1 mM.^22^ The K_m_ for bisulfide was 3 μM and 16 μM for PA2709 and PA0932, respectively (Tables 2 and S2), in line with literature data,^22^^,^^64^ even though the reports on K_m_ values for this substrate are scarce, probably due to the technical difficulties of its determination. The K_m_ for OAS was determined using saturating concentrations of bisulfide and, while the K_m_ for PA2709 (1.1 mM) is in line with the one reported for CysK from *S.* Typhimurium,^22^ the K_m_ of PA0932 (9 mM) is higher (Table S2). In the case of CysK the two k_cat_ values determined by fitting the two dependences are in very good agreement (about 100 s^−1^), while the k_cat_ values calculated for PA0932 are higher when fitting the dependence of initial rate on OAS concentration, likely because 10 mM OAS is not saturating for this enzyme. We thus repeated the measurement of initial rate as a function of HS^−^ concentration at 90 mM OAS and found an apparent K_m_ for bisulfide of 56 μM and a k_cat_ of 797 s^−1^ (Table S2). Being impossible to collect data at saturating bisulfide concentrations, due to linearity limits of the electrode, we performed a multiple fitting of data in Figure 6B and obtained the catalytic parameters of PA0932 for the OAS/bisulfide couple (Table 2). Larger K_m_ values for both OAS and bisulfide are accompanied by a significant increase of k_cat_, with an overall small (about 2-fold) effect on catalytic efficiency. The catalytic parameters for thiosulfate incorporation in *S*-sulfocysteine catalyzed by PA0932 were determined using the discontinuous, ninhydrin-based assay. The low K_m_ for thiosulfate (*vide infra*), combined with the detection limit of the assay, hindered the acquisition of accurate kinetic data. To enhance the accuracy of the calculated parameters, a global fitting was performed on the dependences of reaction rate on OAS concentration collected across multiple thiosulfate concentrations (Figure 6C). The kinetic constants are reported in Table 2. The K_m_ for OAS is 5-fold lower than that measured using bisulfide as second substrate, *i.e.*, 2.7 mM. The K_m_ for thiosulfate is about 160 μM, and the overall efficiency is 15-fold lower than the one for bisulfide. Thiosulfate also displays substrate inhibition, with a K_i_ of 970 μM.

**Table 2:** Kinetic parameters for the formation of L-Cys by PA2709 and PA0932 calculated on data shown in Figure 6 at 25 °C, pH 7

Enzyme	Substrate		K_m,A_ (mM)	K_m,B_ (μM)	k_cat_ (s^−1^)	k_cat_/K_m,A_ (M^−1^ · s^−1^) ·10^5^	k_cat_/K_m,B_ (M^−1^ · s^−1^) ·10^5^	K_i,B_ (mM)
	A	B						
PA2709	OAS	Bisulfide	1.1 ± 0.1	3.1 ± 0.6	102 ± 3^a^ (100 ± 4)^b^	0.90 ± 0.09	330 ± 60	/
PA0932	OAS	Bisulfide	12 ± 3	74 ± 15	1069 ± 123	0.89 ± 0.25	145 ± 34	/
	OAS	Thiosulfate	2.7 ± 0.6	160 ± 50	209 ± 25	0.79 ± 0.20	13 ± 4	0.97 ± 0.83

Kinetic parameters for PA2709 were determined by fitting the single dependences of initial rates on one substrate, while keeping the other constant, to Equation 3. For PA0932 the kinetic parameters were obtained by a global fitting of initial rates with Equations 4 and 5, respectively

a Value calculated from the dependence of initial velocity on OAS concentration.

b Value calculated from the dependence of initial velocity on bisulfide concentration.

### Investigation on the physiological role of CysK and CysM in *P. aeruginosa*

**Figure 7:**
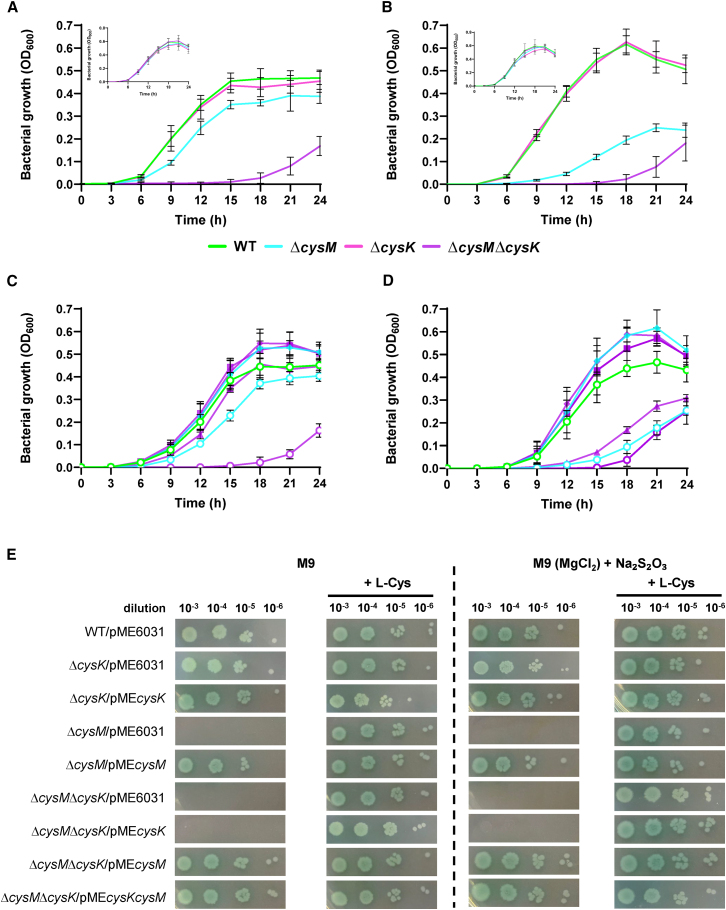
Phenotypic characterization of *P. aeruginosa* Δ*cysM*, Δ*cysK*, and Δ*cysM*Δ*cysK* mutant strains *P. aeruginosa* PAO1 and its isogenic Δ*cysM*, Δ*cysK*, and Δ*cysM*Δ*cysK* mutants were cultured in liquid M9 (A and C) or M9 containing thiosulfate (0.5 mM) as the alternative S-source (B and D). Growth in media supplemented with L-Cys (40 μg/mL) is shown in A and B insets. WT (green), Δ*cysM* (light blue), Δ*cysK* (pink), and Δ*cysM*Δ*cysK* (purple). Circles in C and D indicate the presence of the empty plasmid pME6031, while filled symbols indicate the presence of the pME*cysM* (diamond), pME*cysK* (triangle), pME*cysKcysM* (square), respectively. Growth was measured by turbidimetry (OD_600_) over time. Each value is the average of three biological replicates each performed in duplicate ± standard deviation. Colony growth (E) of *P. aeruginosa* PAO1 (WT) and its isogenic mutants carrying the empty plasmid pME6031 or pME*cysM*, pME*cysK*, pME*cysKcysM*, as indicated. Strains were grown on solid M9 or M9 (MgCl_2_) + Na_2_S_2_O_3_ supplemented or not with L-Cys. Stationary-phase cultures were normalized to OD_600_ = 1, and 5 μL of the 10^−3^ to 10^−7^ dilutions were spotted onto the plates, which were then incubated for 24 h at 37 °C. The images are representative of three independent experiments with similar results.

To investigate the physiological role of the two cysteine synthases isoforms (*i.e.,* CysK and CysM), *cysM*, *cysK*, and *cysKcysM* in-frame deletion mutants have been generated in *P. aeruginosa* PAO1, giving strains *P. aeruginosa* Δ*cysM*, Δ*cysK*, and Δ*cysM*Δ*cysK*, respectively Table 3. Then, the ability of these strains to grow in a chemically defined medium (i.e., M9) with sulfate as the sole S-source has been initially investigated (Figure 7A). No appreciable difference was observed for the single mutant *P. aeruginosa* Δ*cysK* when compared with its parental strain, whereas the *P. aeruginosa* Δ*cysM* mutant displayed a slight growth defect (Figure 7A). Interestingly, the double mutant *P. aeruginosa* Δ*cysM*Δ*cysK* failed to grow for up to 18 h post inoculation, exhibiting only a slight recovery in growth thereafter. To confirm that the observed growth impairment was due to the inability to utilize sulfate, all strains were grown in the same medium supplemented with L-Cys (Figure 7A, inset). Under these conditions, all mutants exhibited a growth profile comparable with that of the wild type (WT), thus confirming the involvement of CysK and CysM in the RSAP in *P. aeruginosa.*

**Table 3:** Bacterial strains and plasmids used in this study

Strain or plasmid	Genotype and/or relevant characteristics	References or source
Strains		
*P. aeruginosa* strains		
PAO1	ATCC 15692 (wild type, prototroph)	American Type Culture Collection
*cysM* mutant	PAO1 Δ*cysM*	This study
*cysK* mutant	PAO1 Δ*cysK*	This study
*cysMcysK* mutant	PAO1 Δ*cysM*Δ*cysK*	This study
*E. coli* strains		
DH5α	*recA1 endA1 hsdR17 supE44 thi-1 gyrA96 relA1* Δ(*lacZYA-argF*)U169 (φ80d*lacZ*ΔM15) F^−^, Nal^R^	Sambrook et al.^103^
HB101	*proA2 hsdS20* (r_b_ m_b_) *recA13 ara-14 lacY1 galK2 rpsL20 supE44 xyl-5 mtl-1* F^*−*^	Sambrook et al.^103^

Plasmids		

pRK2013	Helper plasmid; Tra^+^ Km^R^	Ditta et al.^104^
pME3087	Suicide vector for allelic replacement; ColE1 replicon, Tc^R^	Voisard et al.^105^
pBluescript II SK^−^ (pBS)	Cloning vector, ColE1 replicon, Ap^R^	Stratagene
pBSΔ*cysM*	pBS carrying a 1,975 bp deletion in the *cysM* gene, Ap^R^	This study
pBSΔ*cysK*	pBS carrying a 1,754 bp deletion in the *cysK* gene, Ap^R^	This study
pMEΔ*cysM*	Suicide construct used for the deletion of the *cysM* gene; Tc^R^	This study
pMEΔ*cysK*	Suicide construct used for the deletion of the *cysK* gene; Tc^R^	This study
pME6031	Broad host range plasmid; Tc^R^	Heeb et al.^106^
pME*cysM*	pME6031 derivative carrying the coding sequence of *cysM* with its own promoter	This study
pME*cysK*	pME6031 derivative carrying the coding sequence of *cysK* with its own promoter	This study
pME*cysKcysM*	pME6031 derivative carrying the coding sequences of *cysK* and *cysM* with their own promoters	This study

Since we found that CysM was able to use thiosulfate as an alternative S-source (Figures 5 and 6), we have investigated the growth of *P. aeruginosa* Δ*cysM* in M9, by replacing MgSO_4_ with thiosulfate (Figure 7B) and supplementing the medium with an equimolar concentration of MgCl_2_.

Notably, the growth defect of the *P. aeruginosa* Δ*cysM* mutant was much more pronounced than when grown on sulfate, while all other strains exhibited comparable growth kinetics (Figure 7B). In line with what was previously observed, the growth of *P. aeruginosa* Δ*cysM* and Δ*cysM*Δ*cysK* mutants was rescued by the addition of L-Cys (Figure 7B, inset). No growth was observed for all strains in M9 containing MgCl_2_ and no S-source (data not shown).

As a further validation, *P. aeruginosa* Δ*cysM* and Δ*cysM*Δ*cysK* mutants were genetically complemented by providing *cysM* or both *cysM* and *cysK in trans* on plasmids pME*cysM* and pME*cysKcysM*, respectively (Figures 7C and 7D). The WT phenotype was restored in both *P. aeruginosa* Δ*cysM*/pME*cysM* and Δ*cysM*Δ*cysK*/pME*cysKcysM* strains, while the presence of the empty plasmid pME6031 did not alter the growth behavior of all tested strains (compare Figures 7A and 7B and 7C-D). To better characterize the role of each cysteine synthase in *P. aeruginosa*, the Δ*cysM*Δ*cysK* double mutant was complemented with either *cysM* or *cysK*. Interestingly, *P. aeruginosa* Δ*cysM*Δ*cysK*/pME*cysM* behaved like the WT strain, while the growth of *P. aeruginosa* Δ*cysM*Δ*cysK*/pME*cysK* resembled that of the Δ*cysM* mutant on thiosulfate (Figure 7D), thus confirming the substrate preference of the CysK and CysM enzymes.

In parallel, we also assessed the colony growth of the WT and all mutants, on the above-mentioned media solidified with 1.5% agar (Figure 7E). All strains behaved as in liquid media, except for strains lacking CysM (*i.e.,* Δ*cysM*/pME6031 and Δ*cysM*Δ*cysK*/pME*cysK*), where the growth defect observed in the absence of L-Cys was much more pronounced. Indeed, these strains did not grow on solid media (either with sulfate or thiosulfate) up to 24 h post inoculation. Growth of CysM-deficient strains relapsed after a further 24 h incubation, while the double mutant *P. aeruginosa* Δ*cysM*Δ*cysK*/pME6031 displayed an L-Cys auxotrophy (Figure S7).

## Discussion

Bacterial sulfur assimilation through RSAP occurs *via* a multi-step branched pathway that represents an example of evolutionary divergence, as well as enzymatic redundancy. The last step of RSAP (*i.e.*, cysteine biosynthesis) is catalyzed by cysteine synthases, enzymes that share high sequence similarity across and within microorganisms but also display interesting functional differences. These enzymes are generally classified into two groups based on the substrate specificity (*i.e.,* bisulfide or both bisulfide and thiosulfate) and are usually annotated as OASS-A (CysK) and OASS-B (CysM), respectively. Usually, CysK and CysM isoforms coexist within the same bacterial species, likely to fulfill the need to survive under varying habitats (bisulfide or thiosulfate rich) or for stress adaptation, and are often expressed in response to different environmental stimuli.^68^^,^^69^ Interestingly, some bacterial species only possess one cysteine synthase isoform, which is often indicated among essential genes in the reference species.^70^^,^^71^^,^^72^ Where CysK and CysM isoforms co-occur within the same bacterium, different phenotypes have been described. Deletion of either gene did not markedly affect growth in *S.* Typhimurium, likely due to compensatory activity from the remaining synthase,^36^^,^^68^ while a *cysKcysM* double mutant displayed cysteine auxotrophy, accordingly.^68^ Interestingly, *E. coli* possesses both isoforms, but while *cysM* mutants grew well on a medium lacking cysteine,^73^ CysK seemed to be essential for the growth with sulfate as sole S-source, at least in solid media.^73^^,^^74^^,^^75^^,^^76^ Finally, a non-essential role for these enzymes is suggested in many bacteria due to the presence of multiple isoforms, like in *M. tuberculosis*, where three cysteine synthases exist. Also in this case, the redundant activity did not lead to cysteine auxotrophy for the single mutations, but CysM was shown to serve as the key enzyme for cysteine biosynthesis during *M. tuberculosis* dormancy and to play a critical role in supporting redox homeostasis within host macrophages.^40^

In this study, we have investigated for the first time the presence of cysteine synthases in *P. aeruginosa* and identified four putative OASS enzymes (Table 1). The two candidates exhibiting the highest sequence similarity to *E. coli* CysK and CysM have been selected and characterized.

We have confirmed that both PA2709 and PA0932 are PLP-dependent enzymes, as suggested by the high sequence identity of the active site residues with those of validated cysteine synthases (Figure 2). The two proteins assemble in dimers (Figure 3), as consistently observed for all cysteine synthases characterized so far.^16^ Both enzymes use OAS as the first substrate, with no activity with OPS in the presence of either bisulfide or thiosulfate (Figure 5). While PA2709 can only use bisulfide as nucleophile in the second half-reaction, PA0932 can use both bisulfide and thiosulfate. The catalytic efficiency with thiosulfate is 10-fold lower than with bisulfide yet remains remarkably high (in the 10^6^ M^−1^ s^−1^ range, Table 2). This observation, along with the low K_m_ for thiosulfate, suggests that the reaction is physiologically relevant. Thus, PA2709 can be classified as cysteine synthase/*O*-acetylserine sulfhydrylase (EC 2.5.1.47), while PA0932 is also a *S*-sulfocysteine synthase (*O*-acetyl-L-serine-dependent) (EC 2.5.1.144). In accordance with the accepted nomenclature for these enzymes in *E. coli* and *S.* Typhimurium, PA2709 will hereafter be referred to as PaCysK and PA0932 as PaCysM. It is worth noting that, likely due to technical and sensitivity problems with standard assays, the K_m_ values of CysK/CysM for bisulfide and thiosulfate have been seldom reported in the literature, making direct comparison challenging. However, the K_m_ for bisulfide of PaCysK is comparable with the one measured on the *S.* Typhimurium^22^ and *E. coli*^64^ orthologs, while the K_m_ for thiosulfate is very different with respect to the one reported by Nakamura and colleagues for *S.* Typhimurium CysM (2.7 mM^77^), but is in very good agreement with the one reported for *M. tuberculosis* CysK2 (a OPS-dependent *S*-sulfocysteine synthase), which is 43 μM.^63^ Considering that the concentration of thiosulfate in yeast has been reported to be 900 μM,^78^ the K_m_ of PaCysM is within the physiological concentration of this metabolite. The concentration of OAS in bacterial cells ranges from 200 μM to 20 μM depending on the growth conditions^79^ while the concentration of bisulfide in *E. coli* cells has been estimated in the pM range^80^; thus, both isoforms work under k_cat_/K_m_ regime in the cell and are able to adapt their activity to the availability of substrates. The catalytic efficiency with bisulfide (close to the catalytic perfection) has thus likely evolved to compensate for the low bisulfide concentration in the cells. The co-existence of two enzymes that act on the same substrates (OAS/bisulfide) with comparable efficiency suggests that under physiological conditions they are not expressed at the same time but rather have evolved to face different environmental conditions. It should be kept in mind that, in *E. coli*, *S.* Typhimurium, other bacteria, and plants, CysK can form a bienzymatic complex with CysE where its catalytic activity is reduced by 95%.^28^ Under conditions where most of CysK is engaged within the complex, which are still unexplored in the scientific literature, CysM might play a major role in the biosynthesis of cysteine using bisulfide. Furthermore, PaCysM is also able to use thiosulfate, *i.e.*, a more reduced source of inorganic sulfur with respect to sulfate. The pathway that uses thiosulfate is energetically favorable, because it bypasses the sulfate reduction, saving ATP and NADPH (Scheme 1). Thiosulfate is known to be relatively abundant within the mammalian gut, where it is generated by the host cells as a detoxifying product from microbiota-derived hydrogen sulfide (H_2_S).^81^ Indeed, CysM from *S.* Typhimurium could be active during intestinal infections where the availability of oxygen is low, and the presence of thiosulfate also favors the growth of the bacterium that can use tetrathionate (a derivative of thiosulfate) for respiration.^82^ Interestingly, H_2_S has been reported to be produced by respiratory cells, suggesting that it may also be converted in the lung to thiosulfate, potentially providing a sulfur source for cysteine biosynthesis by respiratory pathogens.^83^ Finally, *S*-sulfocysteine has been proposed in *M. tuberculosis* as a signal for oxidative stress^63^; however, no further evidence supporting this hypothesis has been reported. One distinctive property of PaCysM is its remarkably high thermic stability. The T_m_ of PaCysM (74°C) is significantly higher than that for a mesophilic enzyme (mean T_m_ value of about 62°C^84^) approaching those usually observed for thermophilic enzymes (mean T_m_ value of about 86°C^84^). This finding might suggest a role for PaCysM during stringent response that is associated with harsher intracellular conditions and the activation of proteases (*vide infra*). In line with this hypothesis, Mino and collaborators demonstrated that formation of the CysK-CysE complex protects CysE from proteolytic degradation^85^ and thus suggested a “chaperone” role for CysK. Since within the complex CysK is strongly inhibited, CysM might be required to sustain cysteine biosynthesis under conditions that favor complex formation.

To get further insights into the roles of PaCysK and PaCysM in *P. aeruginosa* and to confirm their substrate specificities, deletion mutants of the corresponding gene(s) (*i.e.,* Δ*cysM*, Δ*cysK*, and Δ*cysM*Δ*cysK*) were generated. Single and double gene deletions revealed that the growth of *P. aeruginosa* Δ*cysK* was comparable with that of the WT under all tested conditions, while *P. aeruginosa* Δ*cysM* showed a slight reduction when grown on sulfate (Figures 7A and 7C). The growth impairment became even more pronounced when thiosulfate was provided as the sole S-source, although the *cysM* deletion did not completely abrogate *P. aeruginosa* growth under these conditions (Figures 7B and 7D). The results suggest that the presence of either one enzyme is sufficient to support the growth of single mutants, in line with what was described in other bacterial species.^68^^,^^86^ Given that PaCysK is unable to use thiosulfate as a S donor (Figure 5), this finding suggests the involvement of an additional CysM-like cysteine synthase (*i.e.*, PA2104 and/or PA1061, Table 1) in supporting growth. This hypothesis is further reinforced by the fact that a double *P. aeruginosa* Δ*cysM*Δ*cysK* mutant failed to grow for up to 18 h post inoculation but exhibited growth recovery at later time points (Figure 7). To confirm the observed phenotypes, the Δ*cysM*Δ*cysK* double mutant was complemented with either *cysM* or *cysK*. It is interesting to highlight that *P. aeruginosa* Δ*cysM*Δ*cysK*/pME*cysK* displayed the same growth behavior of the Δ*cysM* mutant in all tested conditions (Figures 7C and 7D).

Surprisingly, when cultured in solid media (Figure 7E), *P. aeruginosa* lacking CysM or both CysM and CysK failed to grow, whereas the Δ*cysK* mutant exhibited growth comparable with the WT. The growth defect of the Δ*cysM* mutant (as well as of the Δ*cysM*Δ*cysK*/pME*cysK* strain) was restored after an additional 24h incubation (Figure S7), remarkably increasing the bradytroph phenotype displayed by *P. aeruginosa* Δ*cysM* grown in liquid media (Figures 7A–AD). Bacterial growth on solid media does not always mirror planktonic one. Cells on solid surfaces are often subjected to increased nutrient limitation and oxygen deprivation, particularly within dense colonies.^87^^,^^88^ CysM has been reported as the main cysteine synthase under anaerobic conditions in other bacterial species, and although this remains to be experimentally confirmed in *P. aeruginosa*, a similar role cannot be excluded.^89^ This could partially explain the altered phenotype observed for Δ*cysM* mutants on agar plates. We also found that CysM is more stable, which may indicate a more prominent role for this isoform under stress conditions. Interestingly, in the *P. aeruginosa* genome, the *cysM* gene is predicted to form an operon with *relA*, which encodes the enzyme responsible for the synthesis of the alarmone (p)ppGpp. This signaling molecule regulates cellular metabolism in response to nutrient deprivation and other environmental stresses. Although no direct evidence links CysM to the (p)ppGpp regulon, their genomic co-localization raises the hypothesis that their expression might be co-regulated by similar environmental cues. Importantly, the genetic environment of PA0932 is poorly conserved across genera (Figure 2), suggesting a specific function of PaCysM in *P. aeruginosa*. Moreover, it cannot be excluded that toxic metabolic intermediates may accumulate in Δ*cysM* mutants and that their limited diffusion in solid agar media could restrict cell growth. A similar phenotype has been reported in *P. aeruginosa fur* mutants, which are known to grow poorly—or not at all—on solid media, while often growing relatively better in liquid culture.^90^^,^^91^ This growth defect is linked to increased oxidative stress caused by iron dysregulation. Without Fur, excess intracellular iron promotes Fenton reactions generating reactive oxygen species that can accumulate locally and impair viability, particularly under aerobic conditions on solid media. Finally, it is noteworthy that *de novo* L-Cys biosynthesis seems to be linked to impaired biofilm formation in other bacterial species. A *Vibrio fischeri cysK* mutant was reported to exhibit a severe wrinkling defect during colony biofilm formation, suggesting that colony morphology on agar is sensitive to cysteine synthase activity and that the mutation may impair functions required for surface-associated growth.^70^ Moreover, thiol starvation has been shown to induce redox-mediated dysregulation of *E. coli* biofilm components, further supporting the idea that cysteine metabolism can influence biofilm formation through multiple pathways in diverse microorganisms.^92^

In summary, we have characterized two isoforms of cysteine synthase of *P. aeruginosa*, that, while displaying some overlapping functional properties (*i.e.*, ability to use the OAS/bisulfide couple, ability to independently sustain growth in cysteine-depleted medium) likely play different roles, some of which may not be associated with cysteine production. Cysteine biosynthesis has been proposed as a potential source of antibiotic targets, and during the last fifteen years many studies dealing with its exploitation have been published.^93^^,^^94^^,^^95^ Many processes linked to cysteine, its metabolites, or the metabolic pathways for its production could in principle be exploited to design effective antibiotics or enhancers of the antibiotic therapy, provided that the complexity, redundancy, and regulation of the pathway is fully understood. The characterization of cysteine biosynthesis in *P. aeruginosa* is still in its early stages, but it already suggests the presence of distinctive features that warrant further in-depth investigation.

### Limitations of the study

Despite the unquestionable interest of cysteine biosynthesis—particularly, but not exclusively, for its potential in the development of new antimicrobials—this pathway remains poorly characterized, if not entirely unexplored, in many clinically relevant bacterial pathogens. The present study focuses on the last step of the pathway in *P. aeruginosa* and is limited to the characterization of the biochemical properties and the functional role of two isoforms of cysteine synthase. These two enzymes represent the predominant isoforms that are expressed under sulfur starvation conditions. However, it is already clear from the results presented here that other enzyme(s) able to produce cysteine are present in *P. aeruginosa*, as witnessed by the recovery of growth in double deleted mutants. A complete picture of the cysteine biosynthetic potential of *P. aeruginosa* will require the characterization of these additional enzymes and of the growth conditions that favor their relative expression.

## Resource availability

### Lead contact

Requests for further information and resources should be directed to and will be fulfilled by the lead contact, Barbara Campanini (barbara.campanini@unipr.it).

### Materials availability

Plasmids and strains generated in this study are available from the lead contact with a completed materials transfer agreement.

### Data and code availability

• All data reported in this paper will be shared by the lead contact upon request.
• This paper does not report original code.
• Any additional information required to reanalyze the data reported in this paper is available from the lead contact upon request.

## Acknowledgments

We thank Omar De Bei for his valuable support with GPC/SEC experiments and data analysis. We thank Luca Ronda for helpful assistance with the setup of the activity assay based on the H_2_S sensor. We thank Gianmarco Mangiaterra for technical assistance with the initial experimental phase of this study.

This work was supported by PRIN 2022 PNRR - De novo L-cysteine biosynthesis in *Pseudomonas aeruginosa*: pathway assessment for novel antibiotic discovery (ENHANCE) - Project Code P20225HFSK - CUP Codes D53D23022580001 (University of Parma) and H53D23011040001 (10.13039/501100010608University of Urbino), funded by the 10.13039/501100000780European Union – 10.13039/100031478NextGenerationEU to B.C. and E.F.

This work has benefited from the framework of the ALIFAR Initiative, funded by the “10.13039/100017336Departments of Excellence” program of the Italian Ministry for University and Research (10.13039/501100021856MUR, 2023–2027).

## Author contributions

R.M., J.M.L.D., and G.S., investigation, formal analysis, and visualization. M.M. and S.H., supervision and conceptualization. R.P. and S.B., resources and conceptualization. B.C. and E.F., conceptualization, supervision, project administration, resources, funding acquisition, and writing of the original draft.

All authors contributed to manuscript writing and revision, approved the final version, and agreed to be accountable for all aspects of the work, ensuring its integrity and accuracy.

## Declaration of interests

The authors declare no competing interests.

## STAR★Methods

### Key resources table

**Table:** 

REAGENT or RESOURCE	SOURCE	IDENTIFIER
**Bacterial and virus strains**		

See Table 3 for bacterial strains		

**Chemicals, peptides, and recombinant proteins**		

Yeast extract	PanReac AppliChem	Cat# A1552,1000
Tryptone	PanReac AppliChem	Cat# A1553,1000
Sodium chloride (for protein expression and purification)	PanReac AppliChem	Cat# 131659.1211
Sodium chloride (for M9 preparation)	Sigma-Aldrich	Cat# 746398
Phosphate buffered saline	Sigma-Aldrich	Cat# P4417-50TAB
D-(+)-Glucose	Sigma Life Science	Cat# G8270-10 KG
Isopropil β-D-1-tiogalattopiranoside, IPTG, Isopropil β-D-tiogalattoside (IPTG)	Apollo Scientific	Cat# BIMB1008
Phenylmethanesulphonyl fluoride (PMSF)	Apollo Scientific	Cat# PC6222M
Benzamidine	Fluka	Cat# 12072
Pepstatin A	PanReac AppliChem	Cat# A2205,0010
Pyridoxal-5′-phosphate hydrate (PLP)	Sigma-Aldrich	Cat# P9255-5G
Lysozime from chicken egg white	Sigma-Aldrich	Cat# 62971-10G-F
Ethylenediaminetetraacetic acid (EDTA) disodium salt 2-hydrate	PanReac AppliChem	Cat# 131669.1210
Sodium phosphate monobasic	Sigma-Aldrich	Cat# 71496-1 KG
Sodium phosphate dibasic (for protein purification)	Sigma-Aldrich	Cat# 71640-1 KG
Sodium phosphate dibasic dihydrate (for M9 preparation)	Sigma-Aldrich	Cat# 71643
Tris(2-carboxyethyl)phosphine Hydrochloride (TCEP)	Apollo Scientific	Cat# BIT0122
Imidazole	PanReac AppliChem	Cat# A1073,0500
HEPES	PanReac AppliChem	Cat# A1069,0500
Sodium hydroxide	PanReac AppliChem	Cat# 141929.1211
Potassium phosphate dibasic	Sigma-Aldrich	Cat# P3786-1 KG
Potassium dihydrogen phosphate	ACEF	Cat# 001191-1
Bovine serum albumin	Sigma-Aldrich	Cat# A6003-25G
Sodium sulfide	Sigma-Aldrich	Cat# 407410-10G
Hydrochloric acid 37%	PanReac AppliChem	Cat# 131020.1212
Acetic acid glacial	VWR BDH Chemicals	Cat# 20104.334
Ninhydrin	PanReac AppliChem	Cat# A0902,0100
Ethanol 96%	Vener	–
Sodium thiosulfate anhydrous (for enzymatic assays)	Fluka	Cat# 72049
Sodium thiosulfate pentahydrate (for media supplementation)	Sigma-Aldrich	Cat# 217247
*O*-acetyl-L-serine hydrochloride	Sigma-Aldrich	Cat# A6262-5G
*O*-phospho-L-serine	Sigma-Aldrich	Cat# P0878-5G
L-cysteine (for enzymatic assays)	Sigma	Cat# C-7755
L-cysteine hydrochloride monohydrate (for media supplementation)	Fluka Sigma-Aldrich	Cat# 30130
L-serine	Sigma-Aldrich	Cat# 84959-25G
Luria-Bertani (LB) broth	Liofilchem S.r.l	Cat# 610084
Agar (for protein expression)	PanReac AppliChem	Cat# A3477,0500
Agar (for solid M9 preparation)	Liofilchem S.r.l	Cat# 611001
Succinic acid disodium salt	Sigma-Aldrich	Cat# 224731
Magnesium sulfate heptahydrate	Merck	Cat# 1058860500
Magnesium chloride anhydrous	BDH Limited Poole England	Cat# 26123
Ammonium chloride	Merck	Cat# 1011451000
Calcium chloride dihydrate	Sigma-Aldrich	Cat# C3881-500G; Lot# 018K0680
Ampicillin sodium salt	Sigma-Aldrich	Cat# A9518; Lot# BCBZ9179
Nalidixic acid	Sigma-Aldrich	Cat# N8878-5G; Lot# 100K0122
Chloramphenicol	Sigma-Aldrich	Cat# C0378-25G; Lot# 0000127603
Tetracycline hydrochloride	Sigma-Aldrich	Cat# T3383-25G; Lot# 088K0680
Kanamycin sulfate	Sigma-Aldrich	Cat# K4000-25G; Lot# SLBR6873V
Carbenicillin disodium salt	Sigma-Aldrich	Cat# C1389-1G; Lot# 0000097755
PaCysK	This paper	–
PaCysM	This paper	–
Precision Plus Protein™ Unstained Standards	BIO-RAD	Cat# 1610363
Tobacco Etch Virus (TEV) Protease	This paper	–
FastDigest restriction enzyme_XbaI	Thermo Fisher Scientific	Cat# FD0684
FastDigest restriction enzyme_BamHI	Thermo Fisher Scientific	Cat# FD0054
FastDigest restriction enzyme_HindIII	Thermo Fisher Scientific	Cat# FD0504
FastDigest restriction enzyme_KpnI	Thermo Fisher Scientific	Cat# FD0524
FastDigest restriction enzyme_XhoI	Thermo Fisher Scientific	Cat# FD0694
Phusion™ High-Fidelity DNA Polymerase (2 u/μL)	Thermo Fisher Scientific	Cat# F530S
T4 DNA Ligase, 100u	Promega Italia Srl	Cat# M1801
GoTaq(R) G2 DNA Polymerase, 100u	Promega Italia Srl	Cat# M7841

**Critical commercial assays**		

Wizard(R) SV Gel and PCR Clean-up System, 50 preps	Promega Italia Srl	Cat# A9281
PureYield(TM) Plasmid Miniprep System, 100 preps	Promega Italia Srl	Cat# A1223
H_2_S/SULF calibration kit	Unisense A/S	Cat# CALKIT-H2S; Lot# 1.06
Wizard(R) SV Gel and PCR Clean-up System, 50 preps	Promega Italia Srl	Cat# A9281

**Deposited data**		

SMP180 dataset	Abdul-Gader et al.^96^	https://doi.org/10.1093/bioinformatics/btr234
Pseudomonas Genome Database	SciCrunch Registry	RRID:SCR_006590, http://www.pseudomonas.com/

**Oligonucleotides**		

See Table S3 for Oligonucleotides		

**Recombinant DNA**		

pET28a-TEV-PaCysK2709	GenScript, Piscataway, NJ, USA	RRID:SCR_002891, http://www.genscript.com
pET28a-TEV-PaCysK0932	GenScript, Piscataway, NJ, USA	RRID:SCR_002891, http://www.genscript.com

**Software and algorithms**		

SEED	Aziz et al.^97^	RRID:SCR_002129; http://www.theseed.org/wiki/Home_of_the_SEED
geneviewer package (v0.1.10) in R (v4.3.3)	CRAN	https://cran.r-project.org/package=geneviewer
STRING	Szklarczyk et al.^98^	RRID:SCR_005223; http://string.embl.de/
Uniprot	Uniprot Consortium^99^	RRID:SCR_002380; http://www.uniprot.org/
Clustal Omega online (v1.2.4)	Madeira et al.^100^	RRID:SCR_001591; http://www.ebi.ac.uk/Tools/msa/clustalo/
ESPript 3.0	Robert&Gouet^101^	http://espript.ibcp.fr
ProtParam tool	ExPASy Bioinformatics Resource Portal	RRID:SCR_018087; https://doi.org/10.1385/1-59259-584-7:531
DichroWeb	Lobley et al.^57^	https://doi.org/10.1093/bioinformatics/18.1.211
CDSSRT analysis program	Compton et al.^102^	https://doi.org/10.1016/0003-2697(86)90241-1
SigmaPlot 12.0	Grafiti LLC	RRID:SCR_003210; http://www.sigmaplot.com/products/sigmaplot/
GraphPad Prism (v8.0.1)	GraphPad	RRID:SCR_002798; http://www.graphpad.com/
Image Lab (v6.1)	Bio-Rad	RRID:SCR_014210; https://www.bio-rad.com/
SensorTrace Suite (v3.4.700)	Unisense A/S	https://unisense.com/sensortrace-suite/

**Other**		

Amicon Ultra 0.5 mL 10 kDa	Merck Millipore	Cat# UFC501024
Dialysis tubing cellulose membrane	Sigma-Aldrich	Cat# D9527-100FT
Talon SuperFlow metal affinity resin	GE Healthcare	–
H_2_S UniAmp	Unisense A/S	Cat# H2S UNIAMP
H_2_S needle sensor for piercing	Unisense A/S	Cat# SULF-NP
AdvanceBioSec column	Agilent technologies	Cat# PL1180-5301

### Experimental model and study participant details

Bacterial strains and plasmids used in this study are listed in Table 3. Bacteria were routinely grown in Luria-Bertani (LB) broth with good aeration (shaking at 200 rpm) or in LB-Agar. When required, media were supplemented with 40 μg/mL L-Cys. Antibiotics were added to the media at the following concentrations: 100 μg/mL ampicillin (Ap), 20 μg/mL nalidixic acid (Nal), 10 μg/mL chloramphenicol (Cm), 12,5 μg/mL tetracycline (Tc), 25 μg/mL kanamycin (Km) for *E. coli* and 100 μg/mL Tc for *P. aeruginosa*. Bacteria strains were maintained as frozen stock at −80 °C in 20% glycerol.

### Method details

#### Bioinformatic analysis

Gene neighborhood comparisons of PA2709 and PA0932 were performed using the Compare Region Viewer available through the SEED Viewer interface of the RAST (Rapid Annotation using Subsystem Technology) server^97^ and Figure 1 was generated using the geneviewer package (v0.1.10) in R (v4.3.3). To complement the genomic context analysis, predicted functional associations between the protein products of the genes of interest were analyzed using the STRING database.^98^

#### Sequence alignment

Protein sequences of the genes of interest were retrieved from the UniProt database using the reviewed entries.^99^ Multiple sequence alignments were performed using the online version of Clustal Omega online 1.2.4^100^ and displayed with ESPript 3.0.^101^

#### Protein expression and purification

Genes coding for CysK (*cysK*, PA2709) and CysM (*cysM*, PA0932) from *P. aeruginosa* were obtained from the *P. aeruginosa* PAO1 genome databank (https://pseudomonas.com/). Sequences optimized for expression in *E. coli* were synthesized by a gene synthesis service and cloned between NdeI and BamHI restriction sites into the expression vector pET28a-TEV (GenScript, Piscataway, NJ, USA). Both *cysK* and *cysM* genes were synthesized to contain the His-Tag coding sequence at their N-termini, followed by the sequence for the cleavage site for *Tobacco Etch Virus* protease (TEV protease). The proteins were overexpressed in *E. coli* BL21 Tuner host in LB medium containing 50 μg/mL kanamycin and 1% glucose when the cultures reached OD_600_ = 0.6, by adding 1 mM IPTG. After a 4-h induction at 37 °C, cells were harvested by centrifugation. The pellets were resuspended in lysis buffer (50 mM sodium phosphate, 300 mM NaCl, pH 8.0) in the presence of protease inhibitors (0.2 mM PMSF, 0.2 mM benzamidine, 1.5 μM pepstatin A), 1 mM TCEP, 1 mg/mL lysozyme, and 0.2 mM PLP. After a 45-min incubation under agitation at 4 °C, the suspensions were sonicated and then centrifuged to separate the soluble fraction from the debris. Purification was carried out through affinity chromatography using a fast protein liquid chromatography Äkta Prime (GE Healthcare) system, and a Talon SuperFlow resin functionalized with Co^2+^ ions. Supernatants were loaded on the pre-equilibrated cobalt column; the resin was washed with lysis buffer containing 20 mM imidazole and the proteins were eluted using the same buffer containing 300 mM imidazole. The protein-containing fractions were pooled, supplemented with 1 mM EDTA, 1 mM TCEP, TEV at 1:50 (TEV:protein) mass ratio, and PLP at a 1:5 (protein:PLP) molar ratio, and then dialyzed overnight at 4 °C in 10 mM HEPES, pH 8.0. CysK and CysM purification yields were 16 mg/L and 77 mg/L, respectively. The lower yield of CysK is due to the inefficient cleavage of the His_6_-tag using TEV protease. Protein purity was evaluated by SDS-PAGE. Gel image was acquired using a ChemiDoc MP system and densitometric analysis was carried out using Image Lab software (BioRad, Hercules, CA, USA). Samples containing 5 mg of CysK and 3.7 mg of CysM were loaded on the gel together with 1.5-fold and 3-fold dilutions to avoid signal saturation during densitometric analysis. Molecular weight estimation was performed using Precision Plus Protein Unstained Standards (BioRad, Hercules, CA, USA). Protein purity was estimated to be >99% for CysM and 96% for CysK (Figure S8). Small aliquots were flash-frozen in liquid nitrogen and stored at −80 °C until further use.

#### Protein biophysical and functional characterization

If not differently stated, the characterization of PaCysK and PaCysM was carried out in buffer H (100 mM HEPES, pH 7.0).

##### Size exclusion chromatography

Size Exclusion Chromatography was performed on an Agilent GPC/SEC system (Agilent Technologies, Santa Clara, CA, USA) equipped with an AdvanceBio SEC column (300 Å, 2.7 μm, 7.8 × 300 mm; Agilent Technologies, Santa Clara, CA, USA) and a dual-angle light-scattering detector. Both CysK and CysM were loaded at a concentration of 1 mg/mL in buffer H at 20 °C. Flow rate was kept at 1 mL/min. Molecular weight estimation was obtained from light scattering (LS) analysis, using BSA as calibrant.

#### Spectroscopy

Absorption spectra were collected using a Cary4000 spectrophotometer (Agilent Technologies, Santa Clara, CA, USA), fluorescence spectra using a Fluoromax spectrofluorometer (HORIBA Jobin Yvon, Tokyo, Japan). Each spectrum was corrected for the baseline contribution. CysK and CysM concentrations were determined using the extinction coefficients at 280 nm of 18,450 and 24,410 M^−1^ cm^−1^ respectively, calculated with the Expasy tool ProtParam. Absorption spectra were collected at a protein concentration of 20 μM. Emission spectra of the PLP cofactor were collected upon excitation at 330 nm and 412 nm. Emission spectra of tryptophan residues were collected upon excitation at 298 nm, both in the absence and presence of the substrates OAS, OPS, L-Ser or L-Cys. Excitation and emission slits were set to 5 nm for all fluorescence spectra to gain the best signal-to-noise ratio. Substrates were tested at 10 mM.

##### Circular dichroism and thermal stability

The far-UV and near-UV circular dichroism spectra of CysK and CysM were collected using a Jasco 1500 spectropolarimeter (Jasco, Tokyo, Japan) equipped with a Peltier system for temperature control. The far-UV spectra were collected on 4 μM protein solutions in 20 mM potassium phosphate, pH 7.0, 20 °C in a 0.1 cm optical pathlength cuvette. Each spectrum is the average of three acquisitions and was corrected for the buffer contribution. Secondary structure content of CysK and CysM was determined by the deconvolution of CD spectra in the far-UV region with DichroWeb^57^ using the CDSSRT analysis program^102^^,^^107^ and the SMP180 dataset.^96^

The thermal stability of the two proteins was determined by registering the circular dichroism signal at 195 nm in a temperature range of 20°C–100°C under the same conditions, with a data pitch of 1 °C and a digital integration time of 4 s. Melting curves were recorded in duplicate. Data were fitted to Equation 1:^108^

(1) $$\theta =\theta_{0}+\frac{f}{1+e^{-\left(\frac{T-T_{m}}{k}\right)}}$$

where θ is the ellipticity at 195 nm, θ_0_ is an offset, f is the amplitude of the thermal transition, T is the temperature in °C, T_m_ is the melting temperature, and k is the slope. The near-UV circular dichroism spectra of the proteins were collected on 60 μM CysK/CysM solutions in 20 mM potassium phosphate, pH 7.0, at 20 °C in a 0.2 cm optical pathlength cuvette.

#### Activity assays

Two enzymatic assays were used for the determination of the kinetic parameters of CysK and CysM. Kinetic parameters for OAS and bisulfide were determined by monitoring continuously bisulfide depletion due to L-Cys synthesis with an ion selective electrode (Unisense H_2_S microsensor Type I, SULF-NP-403877, Aarhus, Denmark), able to detect hydrogen sulfide.^66^^,^^67^ The SULF microsensor was connected to an *H*_*2*_*S* UniAmp single channel amperemeter and calibrated with the Unisense H_2_S sensor calibration kit. The signal registered by the Unisense SULF microsensor is generated by direct oxidation of hydrogen sulfide on the anode in the tip of the sensor. The sensitivity limit is 0.3 μM H_2_S. Assays were carried out in a 2 mL cuvette equipped with a Teflon cap and a magnetic stirrer. The temperature was maintained at 25 °C by a continuous water flow thermostat system connected to the cuvette housing. Enzymatic assays were conducted in a final volume of 1.5 mL in buffer H, containing a final concentration of either 1.6 or 3.1 nM enzyme, 30 nM BSA, and varying concentrations of OAS and bisulfide ranging from 0.2–90 mM and 1.7–220 μM respectively. Bisulfide was generated directly in the reaction mixture using Na_2_S solutions. The concentration of bisulfide was calculated from the concentration of Na_2_S using a pK_a_ of 7.01 ± 0.02 and pH 7.0.^109^ The reaction was initiated by the addition of the enzymes. Reactions were carried out as follows. The appropriate amount of OAS was added first, and blank was registered after mixing and thermostatting for 120 s. Bisulfide was then added, and the solution was left mixing for another 120 s before beginning to register the signal. Once signal registration started, the reaction mixture was allowed to stand for approximately 100 s to allow the signal to stabilize. The rate of volatilization of hydrogen sulfide was negligible under the assay’s conditions. Then, the enzyme was added with a 10 μL Gastight syringe (# 1701). To determine the kinetic parameters of CysK and CysM, signal expressed in concentration of hydrogen sulfide was first converted into concentration of bisulfide, using Equation 2:^109^

(2) $$\left[{\text{HS}}^{-}\right]=\frac{\left[H_{2}S\right]\;\cdot \;K_{a}}{\left[H^{+}\right]}$$

where [HS^−^], [H_2_S] and [H^+^] are the μM concentrations of bisulfide, hydrogen sulfide and hydrogen ions respectively; and K_a_ is the acid dissociation constant of hydrogen sulfide. Bisulfide concentration was plotted versus time, and linear portions were selected. Initial velocities were obtained by subtracting to the reaction time course the slope of the initial phase. The dependence of the initial rate on the concentration of bisulfide or OAS was then fitted to either Equation 3:

(3) $$v_{0}=\frac{V_{\max}\;\cdot \;\left[S\right]}{K_{m}+\left[S\right]}$$

where v_0_ is the initial velocity, V_max_ is the rate at substrate saturation, [S] is the concentration of either OAS or bisulfide and K_m_ is the respective Michaelis-Menten constant.

For CysM, the dependences of initial velocities on substrates concentration were globally fitted to Equation 4 that accounts for a ping-pong mechanism:^110^

(4) $$v_{0}=\frac{V_{\max}\cdot \left[A\right]\cdot \left[B\right]}{\left(K_{m,B}\cdot \left[A\right]\right)+\left(K_{m,A}\cdot \left[B\right]\right)+\left(\left[A\right]\cdot \left[B\right]\right)}$$

where [A] is the concentration of OAS, [B] is the concentration of bisulfide, K_m,A_ is the Michaelis-Menten constant for OAS and K_m,B_ is the Michaelis-Menten constant for bisulfide.

A colorimetric discontinuous assay based on the reaction of L-Cys with ninhydrin^62^ was employed to initially determine substrate specificity for OAS/OPS and bisulfide/thiosulfate of CysK and CysM and then to determine K_m_ of thiosulfate for CysM. Reactions were conducted in buffer H in 96-well PCR plates thermostatted at 25 °C in a thermoblock, at an enzyme concentration of 3.1 nM. Initial substrate specificity was determined by testing L-Cys formation with four different combinations of substrates: OAS + bisulfide, OAS + thiosulfate, OPS + bisulfide, OPS + thiosulfate. Reaction was initiated by the addition of 0.2 mM sulfide/thiosulfate to a reaction mix containing enzyme, 10 mM OAS/OPS, and 30 nM BSA to prevent enzyme adhesion to well walls. Kinetic parameters for thiosulfate were determined at varying concentrations of OAS ranging from 0.19 to 12.5 mM and a fixed concentration of thiosulfate, ranging from 0.1 to 0.6 mM. Franko’s protocol for 96-well plate format^111^ was followed, with some adjustments. Briefly, aliquots of 30 μL were withdrawn at time intervals, and the reaction was stopped by mixing with 30 μL of acetic acid. 30 μL of ninhydrin were then added to each sample and the mixture was heated at 100 °C for 10 min in a thermal cycler. The solutions were cooled down on the ice and 46 μL were added to the wells of a 96-well plate containing 154 μL of cold ethanol. The absorbance at 550 nm was measured using an Absorbance 96 LED plate reader (Byonoy GmbH, Hamburg, Germany). Time courses were collected at least in duplicate. The amount of L-Cys produced at each time point was calculated from a calibration curve, and data were fitted to a linear equation to calculate the initial rate of L-Cys production. To determine kinetic parameters, the dependence of the initial rate on the concentration of both substrates was fitted to Equation 5 that accounts for a ping-pong mechanism with substrate inhibition for the second substrate:^110^

(5) $$v_{0}=\frac{V_{\max}\cdot \left[A\right]\cdot \left[B\right]}{\left(K_{m,B}\cdot \left[A\right]\right)+\left[K_{m,A}\cdot \left[B\right]\cdot \left(1+\frac{\left[B\right]}{K_{i,B}}\right)\;\right]+\left(\left[A\right]\cdot \left[B\right]\right)}$$

where [A] is the concentration of OAS; [B] is the concentration of thiosulfate; K_m,A_ is the Michaelis-Menten constant for OAS; and K_m,B_ and K_i,B_ are the Michaelis-Menten constant and the inhibition constant for thiosulfate, respectively.

The detection limit of L-Cys of this assay was calculated as the mean of 24 different blanks plus 3 times the standard deviation and was equal to 4.9 μM.

#### Bacterial strains and growth condition

Bacterial strains and plasmids used in this study are listed in Table 3. All media and solutions were prepared with deionized, double-distilled water (ddH_2_O). The ability of *P. aeruginosa* PAO1 and its isogenic Δ*cysM,* Δ*cysK* and Δ*cysM*Δ*cysK* mutants to grow in the presence of different S-sources was investigated by monitoring bacterial growth at 600 nm (OD_600_) over time. Growth assays were performed in the chemically defined minimal medium M9^103^ containing sodium succinate (20 mM) as the C-source. When growth experiments required the alternative presence of thiosulfate (0.5 mM final concentration) as the S-source, MgSO_4_ was replaced by an equimolar concentration of MgCl_2_. Strains were pre-cultured in LB at 37 °C with 200 rpm shaking, then bacterial cells were washed once in M9, diluted to OD_600_ = 0.001 in the appropriate growth medium and dispensed in a 96-well microplate (200 μL/well), to investigate growth over time using a microplate reader (SPARK 10M TECAN, Switzerland).

#### Construction of plasmids for molecular cloning

Oligonucleotides used in this study are listed in Table S3. Standard genetic manipulations were performed according to Sambrook et al.^103^ FastDigest restriction enzymes were purchased from Thermo Fisher Scientific and used in accordance with the instructions provided by the manufacturer.

For the deletion of the *cysK* gene in the *P. aeruginosa* PAO1 chromosome a 868-bp fragment overlapping the ATG of *cysK* and a 904-bp fragment overlapping the TAA of *cysK* were amplified by PCR using the Phusion High-Fidelity DNA Polymerase (2 U/μL) (Thermo Fisher Scientific), the genomic *P. aeruginosa* PAO1 DNA as template and the primer couples *cysK*UPFW/*cysK*UPRV and *cysK*DWFW/*cysK*DWRV, respectively (Table S3). Similarly, to delete the *cysM* gene a 995-bp fragment overlapping the ATG of *cysM* and a 998-bp fragment overlapping the TGA of *cysM* were amplified by PCR using the primer couples *cysM*UPFW/*cysM*UPRV and *cysM*DWFW/*cysM*DWRV, respectively (Table S3). The PCR-amplified upstream and downstream regions of each gene were digested with XbaI-BamHI and BamHI-HindIII, respectively, and then subcloned into the corresponding sites of pBluescript II SK^−^ (pBS), yielding plasmids pBSΔ*cysM* and pBSΔ*cysK*, before being cloned XbaI-HindIII into the final suicide vector pME3087, yielding plasmids pMEΔ*cysM* and pMEΔ*cysK*, respectively. These latter plasmids have then been individually introduced in *P. aeruginosa* PAO1 by triparental mating, using the helper strain *E. coli* HB101/pRK2013, as previously described.^112^ The resulting *P. aeruginosa* strains Δ*cysM* and Δ*cysK* carried an in-frame deletion in the genes of interest. The deletions were confirmed by PCR and subsequent sequencing. Similarly, pMEΔ*cysK* was also introduced in *P. aeruginosa* Δ*cysM*, to generate the double mutant *P. aeruginosa* Δ*cysM*Δ*cysK*.

To complement the *cysM* and *cysK* mutations, DNA fragments containing *cysM* and *cysK* genes with their own promoter regions were PCR amplified from the *P. aeruginosa* PAO1 genome using primer couples *cysM*_compl_FW/*cysM*_compl_RV, and *cysK*_compl_FW/*cysK*_compl_RV, respectively (Table S3). The PCR products were then digested with KpnI and HindIII for *cysM* and XhoI and KpnI for *cysK* and cloned into the corresponding sites of the shuttle vector pME6031, giving pME*cysM* and pME*cysK* plasmids, respectively. These plasmids were then introduced into the corresponding deletion mutants to genetically complement the previously generated mutation.

For the complementation of the double mutant *P. aeruginosa* Δ*cysM*Δ*cysK*, both PCR fragments previously amplified using primer couples *cysM*_compl_FW/*cysM*_compl_RV, and P*cysK_*FW/*cysK*_compl_RV, respectively (Table S3), were digested with KpnI and HindIII (*cysM*) and XhoI and KpnI (*cysK*) and cloned into the corresponding sites of pME6031, yielding plasmid pME*cysKcysM*.

### Quantification and statistical analysis

Enzyme kinetics data are presented as the average of two independent experiments ±standard deviation. Bacterial growth data are presented as the average of three biological replicates each performed in duplicate ±standard deviation.

Kinetics parameters were calculated using SigmaPlot v12.5 (Systat Software, San Jose, CA, USA).

## Supplemental information

Document S1. Figures S1–S8, Tables S1–S3, and supplemental references
