# Supplementary material for: De novo cysteine biosynthesis in Pseudomonas aeruginosa: Characterization of the two main cysteine synthase isoforms
Source: iScience. 2025 Dec 2;29(1):114304. doi: 10.1016/j.isci.2025.114304 (PMC12800425; doi:10.1016/j.isci.2025.114304)
Supplement: Document S1. Figures S1–S8, Tables S1–S3, and supplemental references [file mmc1.pdf]

iScience, Volume *29*

Supplemental information

De novo cysteine biosynthesis in *Pseudomonas aeruginosa*: Characterization of the two main cysteine synthase isoforms

Rebecca Martedì, Jole Maria Lucia D'Angelo, Giulia Sassi, Marialaura Marchetti, Sarah Hijazi, Riccardo Percudani, Stefano Bettati, Barbara Campanini, and Emanuela Frangipani

SUPPLEMENTAL INFORMATION

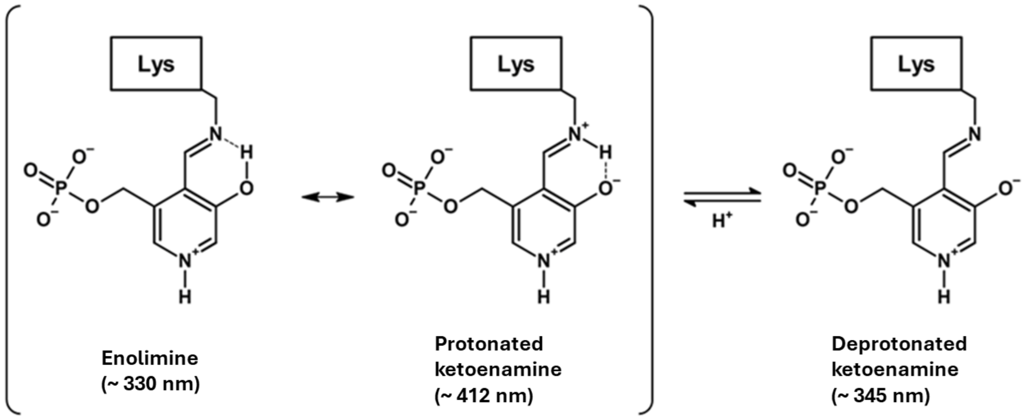

Figure S1. Tautomeric equilibrium between the enolimine and the protonated ketoenamine forms of PLP followed by the deprotonation of ketoenamine tautomer. The wavelength of maximum absorption is indicated in brackets.

	CysK2_Mt	CysM_Mt	CysM_Ec	CysM_St	PA0932	CysK1_Mt	PA2709	CysK_Ec	CysK_St
CysK2_Mt	100.0	30.2	27.4	27.7	29.7	27.7	28.0	24.8	24.8
CysM_Mt	30.2	100.0	40.1	40.1	40.1	39.5	36.3	36.4	36.1
CysM_Ec	27.4	40.1	100.0	94.1	68.8	42.2	41.5	42.0	42.0
CysM_St	27.7	40.1	94.1	100.0	68.8	43.2	42.2	42.3	42.3
PA0932	29.7	40.1	68.8	68.8	100.0	38.6	43.4	40.9	41.2
CysK1_Mt	27.7	39.5	42.2	43.2	38.6	100.0	56.0	58.0	57.3
PA2709	28.0	36.3	41.5	42.2	43.4	56.0	100.0	71.4	71.1
CysK_Ec	24.8	36.4	42.0	42.3	40.9	58.0	71.4	100.0	97.2
CysK_St	24.8	36.1	42.0	42.3	41.2	57.3	71.1	97.2	100.0

Figure S2. Percent identity matrix obtained from the multiple sequence alignment of CysK (PA2709) and CysM (PA0932) protein sequences from *P. aeruginosa*, *E. coli* (Ec), *S.* Typhimurium (St), and *M. tuberculosis* (Mt).

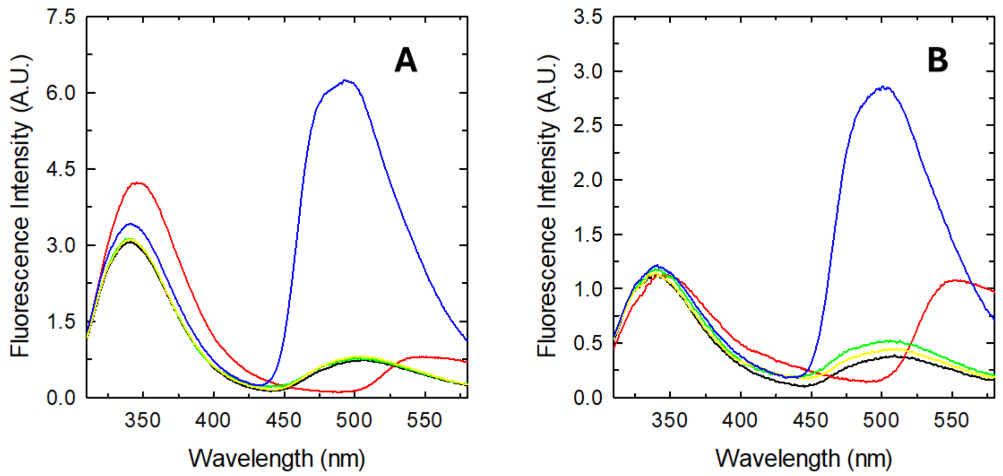

Figure S3. Reaction of PA2709 and PA0932 with potential substrates monitored by fluorescence emission spectroscopy. The fluorescence emission of either 3 µM PA2709 (panel A) or 3 µM PA0932 (panel B) in buffer H was excited at 298 nm in either the absence of ligands (black lines) or in the presence of 10 mM OAS (red lines), 10 mM OPS (green lines), 10 mM L-Ser (yellow lines) and 10 mM L-Cys (blue lines).

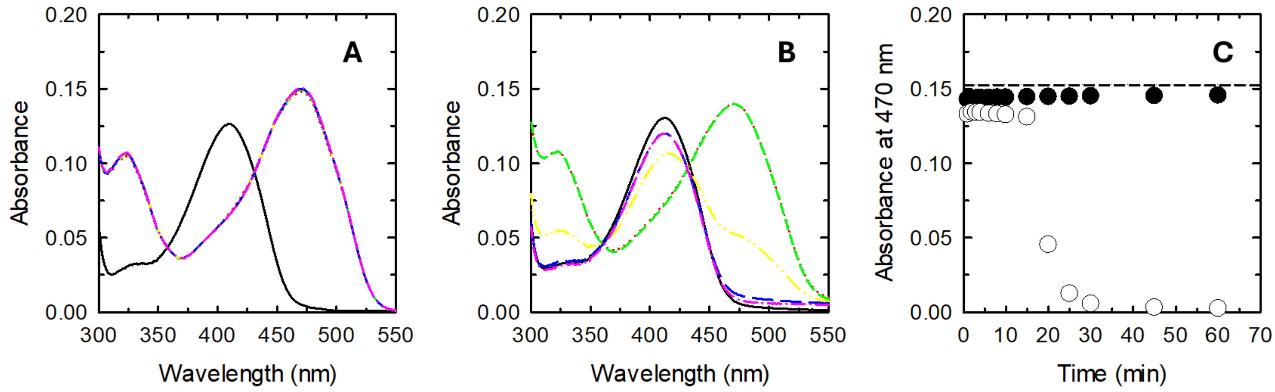

Figure S4. Stability of the α-amino acrylate intermediate formed upon reaction of PA2709 and PA0932 with OAS. A 20 µM solution of either PA2709 (A) or PA0932 (B) (black lines) was added with 100 µM OAS and spectra were taken at 1’ (red dotted lines), 10’ (red/green dashed lines), 20’ (yellow/red dashed lines), 30’ (blue lines) and 60’ (red/pink dashed lines). (C) Absorbance values at 470 nm in panels A and B plotted as a function of time for PA2709 (filled circles) and PA0932 (open circles). The reference top lines refer to the same experiment of panels A and B using 10 mM OAS instead of 100 µM.

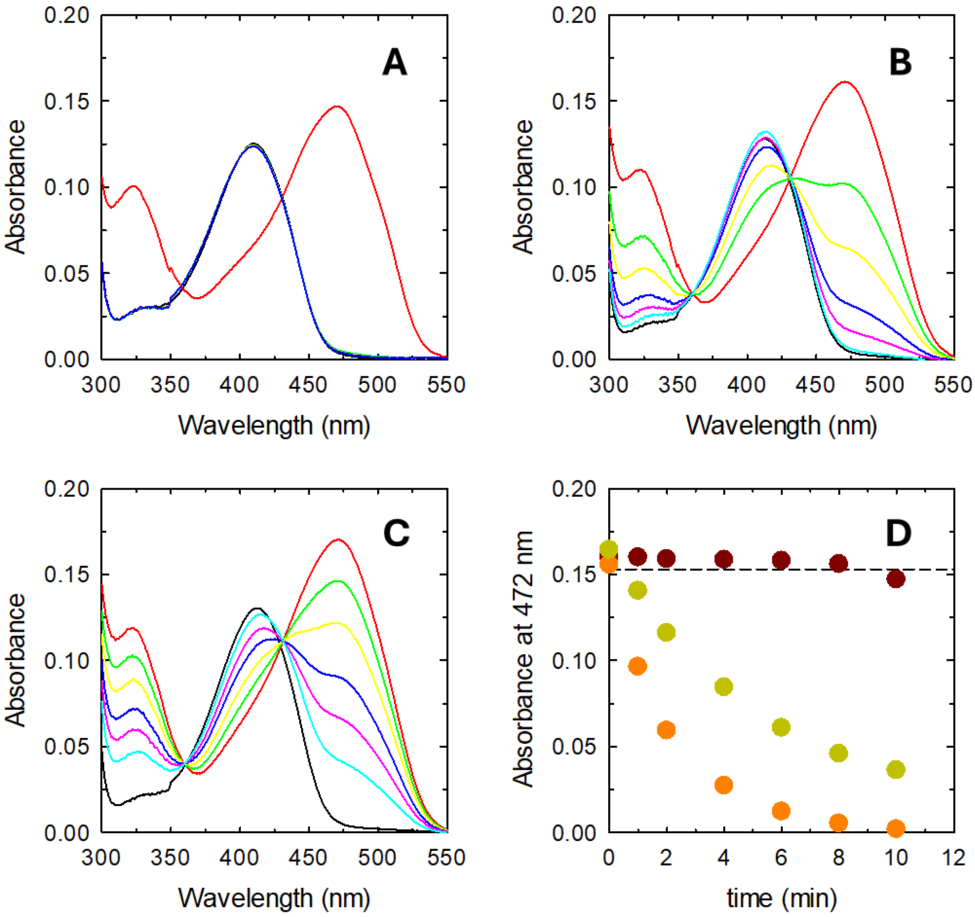

Figure S5. Reactivity of the α-amino acrylate of PA2709 (panel A) and PA0932 (panels B and C) with bisulfide and thiosulfate. Enzyme solutions (black lines) were added of 100 µM OAS (red lines) and of 100 µM of either bisulfide (panels A and B) or thiosulfate (panel C) and the reaction monitored from 1' to 10'. The kinetics at 470 nm for PA0932 in the absence of nucleophiles (red dots) and in the presence of bisulfide (orange dots) or thiosulfate (green dots) are compared (panel D).

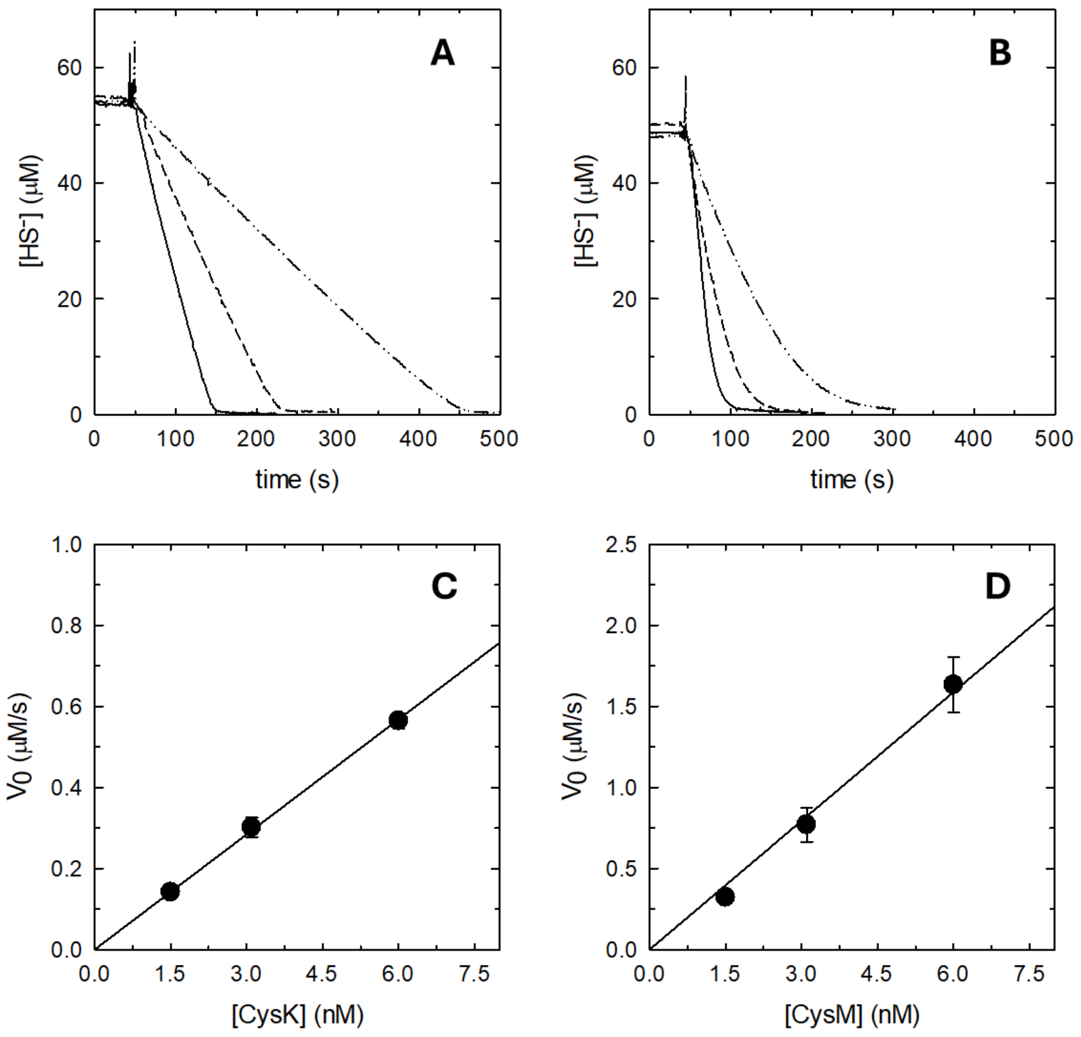

Figure S6. Set-up of a continuous assay to monitor *O*-acetylserine sulfhydrylase activity by a sulfide-selective electrode. (A) kinetic traces of bisulfide consumption by PA2709 at 1.5, 3.1 and 6 nM. (B) kinetic traces of bisulfide consumption by PA0932 at 1.5, 3.1 and 6 nM. (C) dependence of the initial velocity as calculated from kinetic traces in panel A on PA2709 concentration. (D) dependence of the initial velocity as calculated from kinetic traces in panel A on PA0932 concentration. Assays were conducted at 25 °C in buffer H, in the presence of 10 mM OAS and 50 µM bisulfide. Data are the average of two independent experiments ± standard deviation

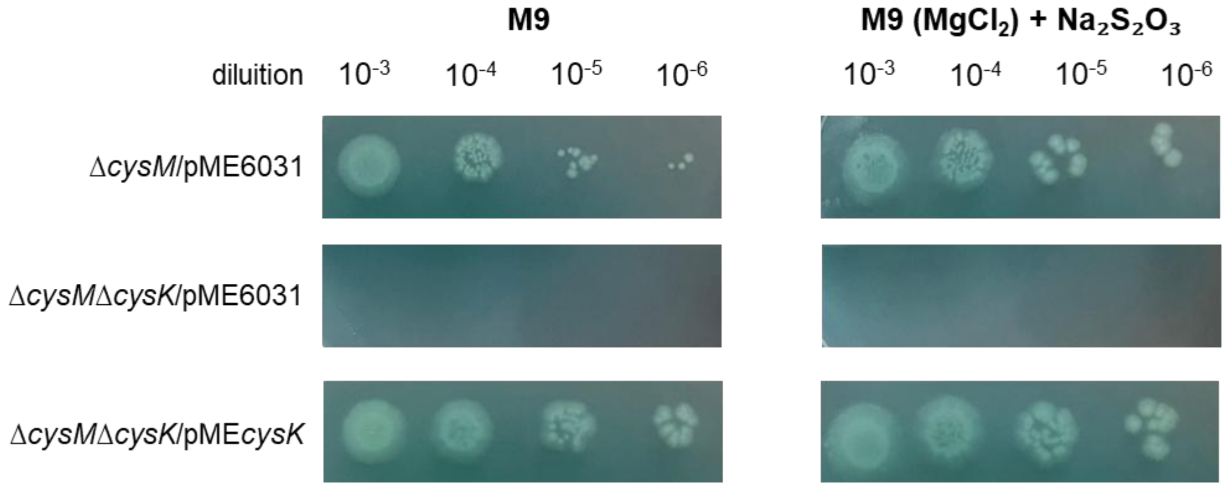

Figure S7. Colony growth of *P. aeruginosa* Δ*cysM* and Δ*cysM*Δ*cysK* mutant strains carrying the empty plasmid pME6031 or pMEcysK, as indicated. Strains were grown on solid M9 or M9 ($MgCl_2$) + $Na_2S_2O_3$. Stationary-phase cultures were normalized to OD_{600}=1, and 5 µL of the 10^{-3} to 10^{-7} dilutions were spotted onto the plates, which were then incubated for 48 h at 37 °C. The images are representative of three independent experiments with similar results.

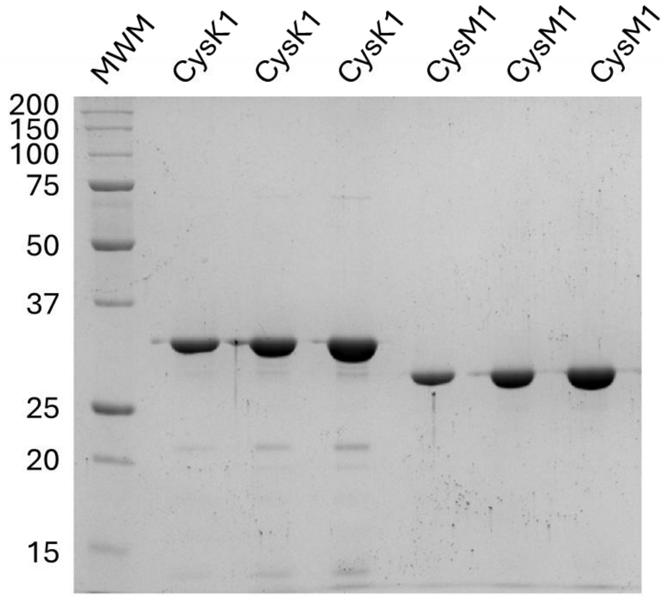

Figure S8. SDS-PAGE of the CysK and CysM preparations used in this study. For each protein preparation three different dilutions of the stock solution were loaded (1-3). Precision Plus Protein™ Unstained Standards (Bio-Rad) were used as molecular weight markers (MWM). Densitometric analysis of the most diluted samples (CysK1 and CysM1) allowed to calculate a purity of 96 % for CysK and >99% for CysM.

Table S1. Secondary structure content of PA2709 and PA0932 as determined by the deconvolution of CD spectra in the far-UV region with DichroWeb[1] using the CDSSRT analysis program[2,3] and the SMP180 dataset.[4]

**Protein**	**% α-helix**	**% β-strands**	**% β-turns**	**% random coil**
PA2709	39	22	11	27
PA0932	32	29	10	29

Table S2. Apparent kinetic parameters for the formation of L-Cys by PA0932 calculated from the fitting of the dependence of initial rates on either OAS concentration in the presence of 160 μM bisulfide or bisulfide concentration in the presence of 10 mM and 90 mM OAS in buffer H, pH 7 at 25 °C using Equation 3.

**$K_{m,OAS}$ (mM)**	**k_{cat} (s^{-1})**	**K_{m,HS^-} (μM)**	**k_{cat} (s^{-1})**
9.1 ± 0.6	499 ± 12	16.0 ± 4.0*<br>56.3 ± 6.0**	333 ± 26*<br>797 ± 31**

*Values calculated from the dependence of initial velocity on bisulfide concentration at 10 mM OAS.
**Values calculated from the dependence of initial velocity on bisulfide concentration at 90 mM OAS.

Table S3. Oligonucleotides used in this study.

Name	Sequence (5' → 3')*	Restriction site	Use
*cysM*UPFW	CGCTCTAGAGCAACCCGAACTACCTG	XbaI	Generation of pMEΔ*cysM*
*cysM*UPRV	CGCGGATCCGGTCATGGAGGCACTCGT	BamHI	Generation of pMEΔ*cysM*
*cysM*DWFW	CGCGGATCCTCCGGCGTCTATGACCCG	BamHI	Generation of pMEΔ*cysM*
*cysM*DWRV	CCCAAGCTTGCCCAGCCCGCAGAACA	HindIII	Generation of pMEΔ*cysM*
*cysK*UPFW	CGCTCTAGAACTTGCCATAGGCGCTG	XbaI	Generation of pMEΔ*cysK*
*cysK*UPRV	CGCGGATCCGCTCATGATGGTGTCCTTGT	BamHI	Generation of pMEΔ*cysK*
*cysK*DWFW	CGCGGATCCCTGACCCAGTAACCACCGA	BamHI	Generation of pMEΔ*cysK*
*cysK*DWRV	CCCAAGCTTCATTGTTGTAGGTGTCCT	HindIII	Generation of pMEΔ*cysK*
*cysM*_compl_FW	CGGGGTACCACTACGTTTCGATGACCACCT	KpnI	Generation of pME*cysM* and pME*cysKcysM*
*cysM*_compl_RV	CCCAAGCTTGCCAACTGCTCGCCGTGTT	HindIII	Generation of pME*cysM* and pME*cysKcysM*
P*cysK*_FW	CCGCTCGAGCCGTTGTCCTTGACGATG	XhoI	Generation of pME*cysK* and pME*cysKcysM*
*cysK*_compl_RV	CGGGGTACCGATTCGATACGCCTGGCGC	KpnI	Generation of pME*cysK* and pME*cysKcysM*

*Restriction sites are underlined.

1 Lobley, A., Whitmore, L. & Wallace, B. A. DICHROWEB: an interactive website for the analysis of protein secondary structure from circular dichroism spectra. *Bioinformatics* **18**, 211-212, doi:10.1093/bioinformatics/18.1.211 (2002).

2 Compton, L. A. & Johnson, W. C. Analysis of protein circular dichroism spectra for secondary structure using a simple matrix multiplication. *Analytical Biochemistry* **155**, 155-167, doi:10.1016/0003-2697(86)90241-1 (1986).

3 Manavalan, P. & Johnson, W. C. Variable selection method improves the prediction of protein secondary structure from circular dichroism spectra. *Analytical Biochemistry* **167**, 76-85, doi:10.1016/0003-2697(87)90135-7 (1987).

4 Abdul-Gader, A., Miles, A. J. & Wallace, B. A. A reference dataset for the analyses of membrane protein secondary structures and transmembrane residuesusing circular dichroism spectroscopy. *Bioinformatics* **27**, 1630-1636, doi:10.1093/bioinformatics/btr234 (2011).
